# The Real‐World Safety Profile of Temazepam: A 20‐Year Pharmacovigilance Analysis Based on the Large‐Scale FAERS Database

**DOI:** 10.1002/cns.70836

**Published:** 2026-03-23

**Authors:** Zujun Wen, Xiang Liu, Peng Liu

**Affiliations:** ^1^ Department of Pharmacy Heyuan People's Hospital Heyuan China; ^2^ Department of Pharmacy The First Affiliated Hospital of Chongqing Medical and Pharmaceutical College, the Sixth People's Hospital of Chongqing Chongqing China; ^3^ Department of Pharmacy Dazhou Central Hospital Dazhou Sichuan China

**Keywords:** adverse events, disproportionality analysis, FARES, insomnia, real‐world pharmacovigilance analysis, temazepam

## Abstract

**Objective:**

Temazepam has not been adequately investigated for its long‐term safety profile in broad patient populations. This study aimed to characterize temazepam's real‐world long‐term safety profile.

**Methods:**

Reports of adverse events from Q1 2006 to Q4 2025 comprising temazepam were collected from the FAERS database. Data were analyzed using disproportionality methods including the proportional reporting ratio (PRR), the reporting odds ratio (ROR), the multi‐item gamma poisson shrinker (MGPS), and the Bayesian confidence propagation neural network (BCPNN).

**Results:**

A total of 2788 adverse event reports associated with temazepam were retrieved. The most frequently reported events were drug ineffective, eye irritation, and toxicity to various agents. We also detected numerous ocular risk signals absent from the product label, together with potential abuse signals. Furthermore, our study identified several behavioral risk signals such as abnormal behavior, aggression, and amnesia and parasomnias‐related adverse events like nightmare and somnambulism.

**Conclusions:**

A comprehensive analysis of the large scale real world FAERS database was performed in this study identifying an extensive spectrum of adverse events associated with temazepam. Our study confirmed established risks and detected potential novel signals including ocular toxicities (e.g., eye irritation, intraocular pressure increased, glaucoma, eye inflammation, and photophobia) as well as neuro behavioral adverse outcomes such as aggression, amnesia and parasomnias (e.g., nightmare and somnambulism). These findings provide compelling support for temazepam clinical monitoring and risk assessment.

## Graphical abstract

This study aimed to comprehensively characterize temazepam's safety profile by a large‐scale analysis of real‐world data. Most adverse events occur within the first 30 days of treatment. The findings highlighted the need for enhanced clinical vigilance and dynamic. Individualized risk–benefit reassessment is imperative for all stakeholders throughout temazepam therapy.

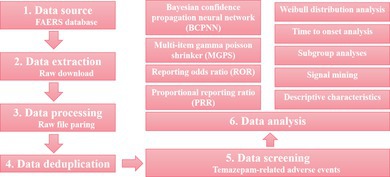

## 1 Introduction

One of the most common and persistent medical disorders, insomnia affects the quality of life for around 30% of the global population and has a significant socioeconomic impact [1, 2]. It manifests as difficulty initiating or maintaining sleep, or as early‐morning awakening with inability to return to sleep. In fact, patients suffering from insomnia may experience significant impairments in their daytime function, including fatigue, excessive sleepiness, impaired cognitive function, affective lability, and impaired concentration [3]. Additionally, insomnia has been identified as an independent risk factor for several non‐communicable conditions, such as depression, cardiovascular disease, hypertension, obesity, and diabetes, which further exacerbate the long‐term impact of insomnia on public health [4]. The principal goals of treating insomnia are to enhance the quality of sleep, lessen waking symptoms, enhance daytime function, and decrease suffering [5]. Cognitive behavioral therapy for insomnia (CBT‐I), due to its improvement in symptoms without drug side effects and continuous efficacy, is often considered the preferred treatment for insomnia [6]; however, limited availability of trained therapists and higher out‐of‐pocket costs often oblige patients to seek drug treatment [7].

Temazepam, a benzodiazepine available since 1981, is approved by the US Food and Drug Administration for short term treatment of insomnia [8]. It acts as a gamma aminobutyric acid positive allosteric modulator, enhancing neuronal inhibition through increased gamma‐aminobutyric acid (GABA) activity [9]. The use of temazepam is still widespread around the world, with high prescription rates in Europe, Australia, and several Asian countries [10, 11, 12, 13]. But the extensive usage of temazepam has also sparked worries about its associated adverse effects. Adverse effects are mainly central nervous system related, including drowsiness, fatigue, and dizziness [14, 15], and may extend to mental health, causing emotional fluctuations and the emergence of fantasies [12, 16]. Meta‐analyses and clinical trials have identified some risks, but were limited by strict inclusion criteria, small samples, short follow‐up, homogeneous individuals, and concentrated adverse events within a single or very few systems. Consequently, the complete safety profile of temazepam in real‐world settings remains uncharacterized.

In drug safety evaluation, real‐world data play a pivotal role and supplement post‐marketing surveillance. The FDA Adverse Event Reporting System (FAERS) is the largest and most comprehensive spontaneous reporting database worldwide. Its high sample sizes and long term monitoring enable early detection of rare or severe adverse event signals. The FAERS has been extensively used for signal detection and pharmacovigilance. Therefore, we utilized the FAERS database to perform a comprehensive analysis of temazepam adverse reactions, aiming to uncover potential risk signals and provide evidence for safer clinical use.

## 2 Materials and Methods

### 2.1 Study Design and Data Sources

**FIGURE 1:**
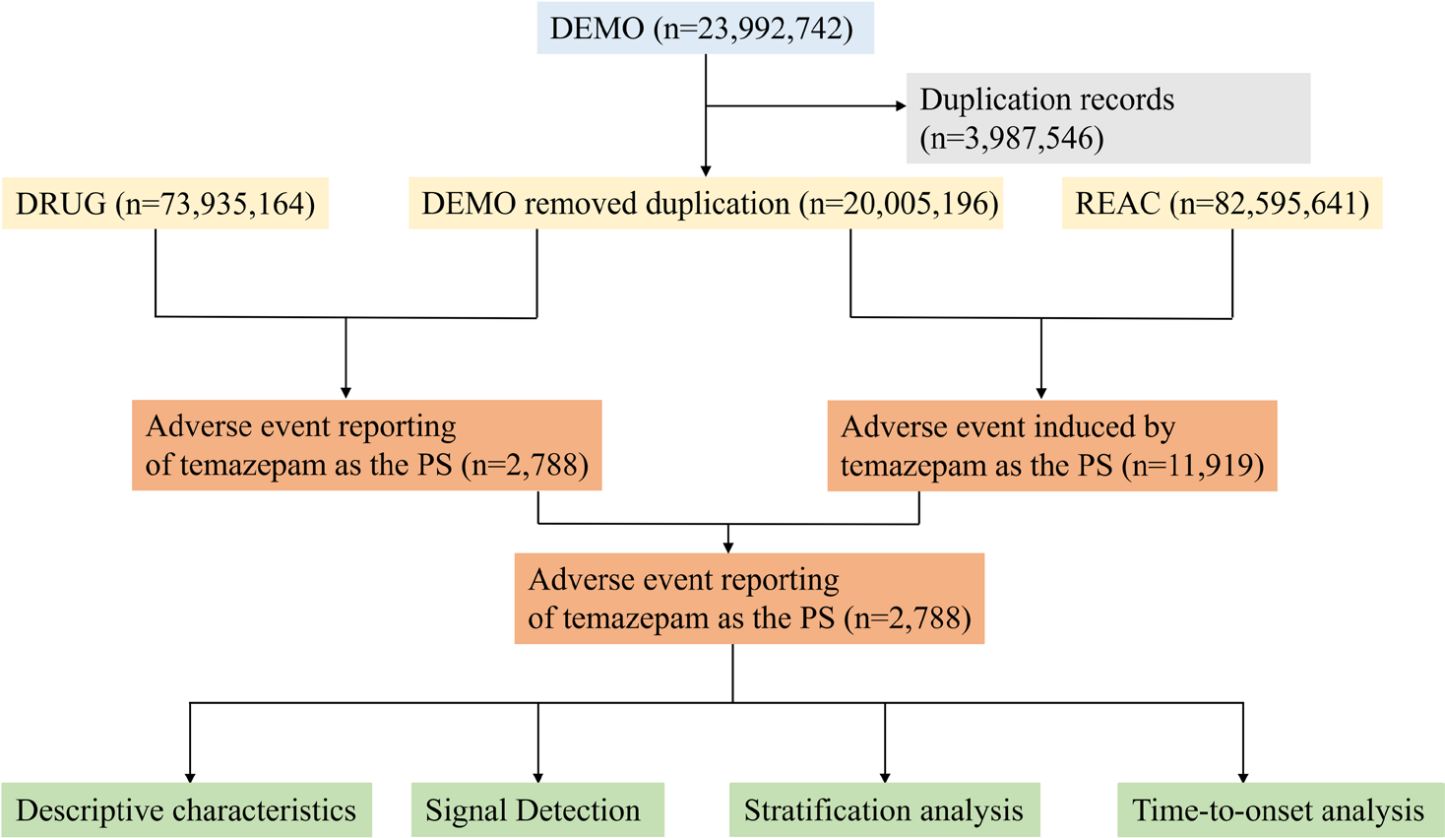
Data collection flow chart for adverse events of temazepam. DEMO, demographic and administrative information; DRUG, drug information; REAC, coded for the adverse events; PS, primary suspect.

The FAERS database compiles adverse event reports submitted voluntarily by consumers, healthcare professionals, and manufacturers and is updated quarterly. We used the FAERS database to conduct a post‐marketing pharmacovigilance study of temazepam and extracted all temazepam related reports from Q1 2006 to Q4 2025. Subsequent data management standardized terminology according to the Medical Dictionary for Regulatory Activities (MedDRA) Preferred Terms (PTs) and System Organ Classes (SOCs) and followed the FDA deduplication guidance: when CASE_ID were identical, the report with the most recent FDA_DT was retained; when PRIMARY_ID were identical, the higher PRIMARY_ID was kept. Figure 1 showed the flowchart.

### 2.2 Statistical Analysis

The most common approach in pharmacovigilance to assess potential drug‐related adverse events is disproportionality analysis. Methods for disproportionality analysis include frequency‐based data mining techniques like the proportional reporting ratio (PRR) and reporting odds ratio (ROR), as well as Bayesian data mining techniques such as the Bayesian Confidence Propagation Neural Network (BCPNN) and the multiple gamma Poisson shrinker (MGPS). Frequency‐based approaches are characterized by high sensitivity, algorithmic simplicity, and intuitive results, making them suitable for initial signal screening in large spontaneous‐report databases, but they manifest lower specificity, greater signal instability, and susceptibility to outliers. By contrast, Bayesian approaches provide excellent signal‐to‐noise ratio control, improving the accuracy and sensitivity of signal detection, and effectively lessen the impact of data sparsity, making them ideal for stable detection of rare adverse event signals. However, they usually have lesser sensitivity and are computationally challenging [17]. To minimize false positive findings, an adverse event was considered a potential temazepam‐related reaction only if it simultaneously met the positivity thresholds of all four methods. Through cross‐validation, this integrative approach reduces false positives while increasing detection efficiency by utilizing the complementary characteristics of each algorithm. Additionally, it makes it greater to identify rare adverse events. It is important to note that disproportionality analysis identifies signals, not causal relationships. Contingency tables and corresponding formulae are given in Tables S1 and S2, respectively. Time to onset was defined as the interval between the start date (START_DT) of temazepam use and the adverse event onset date (EVENT_DT), and all analyses were performed using R software version 4.2.2.

## 3 Results

### 3.1 Clinical Characteristics Description

As shown in Figure 1, there were 20,005,196 unique reports out of the 23,992,742 reports in the DEMO dataset after excluding 3,987,546 duplicates. Among these, 2788 involved temazepam as the primary suspect (PS), and 11,919 adverse events were recorded. Table 1 depicted that female reports predominated (58.7%, *n* = 1637) compared with male reports (27.9%, *n* = 778). The majority of cases occurred in adults aged 18 to 65 years (36.4%). The United States supplied most reports (82.8%), followed by Britain (8.1%) and Germany (1.6%). Consumers submitted 58.3% of reports, and healthcare professionals 37.8%. Report numbers peaked in 2018 and 2019 (each 9.1%).

**TABLE 1:** Clinical characteristics of temazepam adverse event reports from the FAERS database (Q1 2006 to Q4 2025).

Characteristics	Case numbers	Case proportion (%)
Number of events	2788	
**Gender**		
Male	778	27.9%
Female	1637	58.7%
Miss	373	13.4%
**Age**		
< 18	15	0.5%
18–65	1015	36.4%
65–85	692	24.8%
> 85	113	4.1%
Miss	937	34.0%
**Top 5 reported countries**		
United States	2307	82.8%
Britain	227	8.1%
Germany	46	1.6%
Netherlands	43	1.5%
Australia	41	1.5%
**Reporter**		
Healthcare professional	1054	37.8%
Non‐healthcare professional	1624	58.3%
Miss	110	3.9%
**Reported years**		
2006	28	1.0%
2007	31	1.1%
2008	52	1.9%
2009	78	2.8%
2010	131	4.7%
2011	93	3.3%
2012	233	8.4%
2013	81	2.9%
2014	146	5.2%
2015	175	6.3%
2016	212	7.6%
2017	225	8.1%
2018	253	9.1%
2019	252	9.0%
2020	213	7.6%
2021	174	6.2%
2022	119	4.3%
2023	99	3.6%
2024	121	4.3%
2025	72	2.6%

Abbreviation: IQR, interquartile range.

### 3.2 Signal Mining Results

**FIGURE 2:**
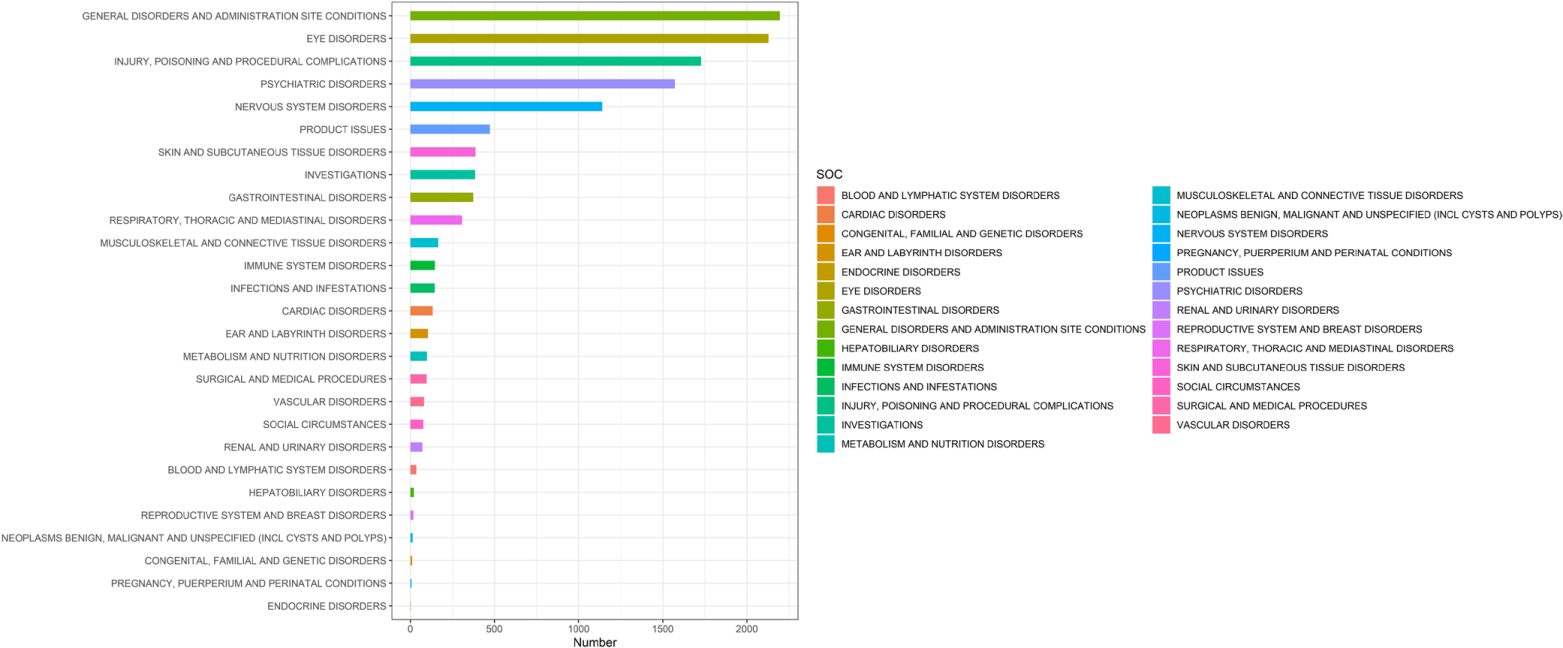
System Organ Classes (SOC) distribution.

Table 2 presented the SOC‐level signal of temazepam‐related adverse events. 27 SOCs were identified (Figure 2). General disorders and administration site conditions (*n* = 2195, ROR 1.05, PRR 1.04, IC 0.06, EBGM 1.04), eye disorders (*n* = 2129, ROR 10.68, PRR 8.95, IC 3.16, EBGM 8.94), injury, poisoning and procedural complications (*n* = 1728, ROR 1.41, PRR 1.35, IC 0.43, EBGM 1.35), psychiatric disorders (*n* = 1573, ROR 2.65, PRR 2.43, IC 1.28, EBGM 2.43), and nervous system disorders (*n* = 1141, ROR 1.16, PRR 1.15, IC 0.20, EBGM 1.15) had the most reports among them. These results suggested the need for greater caution in the potential risks of temazepam‐associated ocular, psychiatric, and neurological adverse events.

**TABLE 2:** Signal strength of temazepam adverse events across System Organ Classes (SOC) in the FAERS database.

SOC	Case numbers	ROR (95% Cl)	PRR (χ^2^)	EBGM (EBGM05)	IC (IC025)
Eye disorders*	2129	10.68 (10.19–11.19)	8.95 (15324.61)	8.94 (8.53)	3.16 (3.09)
Investigations	385	0.53 (0.48–0.58)	0.54 (158.3)	0.54 (0.49)	−0.88 (−1.03)
Product issues*	473	2.42 (2.21–2.66)	2.37 (379.7)	2.37 (2.16)	1.24 (1.1)
Endocrine disorders	2	0.06 (0.02–0.26)	0.07 (26.95)	0.07 (0.02)	−3.94 (−5.07)
Vascular disorders	82	0.33 (0.26–0.4)	0.33 (113.73)	0.33 (0.27)	−1.6 (−1.9)
Social circumstances*	77	1.41 (1.13–1.76)	1.41 (9.07)	1.41 (1.12)	0.49 (0.16)
Hepatobiliary disorders	21	0.2 (0.13–0.3)	0.2 (68.12)	0.2 (0.13)	−2.33 (−2.89)
Nervous system disorders*	1141	1.16 (1.09–1.23)	1.15 (22.89)	1.15 (1.08)	0.2 (0.11)
Immune system disorders	145	1.05 (0.89–1.24)	1.05 (0.35)	1.05 (0.89)	0.07 (−0.17)
Cardiac disorders	132	0.44 (0.37–0.52)	0.45 (92.5)	0.45 (0.38)	−1.16 (−1.41)
Psychiatric disorders*	1573	2.65 (2.51–2.79)	2.43 (1398.23)	2.43 (2.3)	1.28 (1.2)
Musculoskeletal and connective tissue disorders	167	0.26 (0.22–0.3)	0.27 (352.43)	0.27 (0.23)	−1.9 (−2.12)
Congenital, familial and genetic disorders	10	0.28 (0.15–0.53)	0.28 (18.03)	0.28 (0.15)	−1.81 (−2.59)
Neoplasms benign, malignant and unspecified (incl cysts and polyps)	13	0.04 (0.03–0.08)	0.05 (265.09)	0.05 (0.03)	−4.45 (−5.12)
Metabolism and nutrition disorders	99	0.38 (0.31–0.46)	0.38 (99.67)	0.38 (0.32)	−1.38 (−1.66)
Reproductive system and breast disorders	17	0.16 (0.1–0.26)	0.16 (75.12)	0.16 (0.1)	−2.64 (−3.25)
Surgical and medical procedures	97	0.59 (0.49–0.73)	0.6 (26.69)	0.6 (0.49)	−0.74 (−1.03)
General disorders and administration site conditions*	2195	1.05 (1.01–1.1)	1.04 (4.96)	1.04 (1)	0.06 (0)
Injury, poisoning and procedural complications*	1728	1.41 (1.34–1.48)	1.35 (176.52)	1.35 (1.28)	0.43 (0.36)
Blood and lymphatic system disorders	37	0.19 (0.13–0.26)	0.19 (131.28)	0.19 (0.14)	−2.41 (−2.84)
Pregnancy, puerperium and perinatal conditions	6	0.12 (0.05–0.27)	0.12 (38.72)	0.12 (0.05)	−3.05 (−3.95)
Infections and infestations	144	0.22 (0.19–0.26)	0.23 (393.83)	0.23 (0.19)	−2.12 (−2.36)
Gastrointestinal disorders	374	0.35 (0.31–0.38)	0.37 (448.77)	0.37 (0.33)	−1.45 (−1.6)
Ear and labyrinth disorders*	105	2.01 (1.66–2.43)	2 (52.68)	2 (1.65)	1 (0.7)
Renal and urinary disorders	72	0.31 (0.24–0.39)	0.31 (112)	0.31 (0.25)	−1.68 (−2.01)
Skin and subcutaneous tissue disorders	387	0.57 (0.51–0.63)	0.58 (123.7)	0.58 (0.53)	−0.78 (−0.93)
Respiratory, thoracic and mediastinal disorders	308	0.54 (0.48–0.6)	0.55 (119.13)	0.55 (0.49)	−0.86 (−1.03)

*Note:* Asterisks (*) indicate statistically significant signals in algorithm.

Abbreviations: CI, confidence interval; EBGM, empirical Bayesian geometric mean; EBGM05, the lower limit of the 95% CI of EBGM; IC, information component; IC025, the lower limit of the 95% CI of the IC; PRR, proportional reporting ratio; ROR, reporting odds ratio; SOC, System Organ Classes.

At the PT level, temazepam‐related adverse events were ranked by frequency and evaluated for positive signals. The top 50 positive PTs were displayed in Table 3. The five most frequent were drug ineffective, toxicity to various agents, eye irritation, vision blurred, and eye pain. In particular, our analysis also found a notable number of potential ocular risks, such as eye irritation, vision blurred, eye pain, ocular hyperaemia, eye swelling, visual impairment, lacrimation increased, intraocular pressure increased, glaucoma, eye inflammation, photophobia that were absent from the product label except for vision blurred, and require further investigation. Additionally, we detected behavioral risk signals including abnormal behavior, aggression, amnesia, and depressed level of consciousness, together with parasomnia‐related events such as nightmare and somnambulism; these required further investigation. Moreover, adverse events associated with potential abuse risk, including drug abuse, intentional overdose, product residue present, poisoning, and extra dose administered were detected; several showed relatively high signal strength and carried significant clinical implications, warranting heightened vigilance. All positive‐signal events were provided in Table S3.

**TABLE 3:** Top 50 frequency of adverse events at the PT level for temazepam.

PT	Case numbers	ROR (95% CI)	PRR (χ^2^)	EBGM (EBGM05)	IC (IC025)
Drug ineffective*	668	2.63 (2.43–2.84)	2.54 (634.79)	2.53 (2.34)	1.34 (1.22)
Eye irritation*	263	26.1 (23.09–29.5)	25.55 (6185.42)	25.46 (22.52)	4.67 (4.36)
Eye pain*	230	22.59 (19.82–25.74)	22.17 (4639.16)	22.1 (19.4)	4.47 (4.15)
Toxicity to various agents	224	6.19 (5.42–7.06)	6.09 (954.83)	6.08 (5.33)	2.61 (2.38)
Vision blurred	221	8.63 (7.55–9.86)	8.49 (1461.18)	8.48 (7.42)	3.08 (2.84)
Insomnia*	213	4.12 (3.6–4.72)	4.07 (494.77)	4.07 (3.55)	2.02 (1.8)
Completed suicide	208	13.64 (11.89–15.65)	13.42 (2389.4)	13.4 (11.68)	3.74 (3.46)
Ocular hyperaemia*	155	17.75 (15.15–20.81)	17.54 (2412.57)	17.49 (14.93)	4.13 (3.75)
Product use in unapproved indication	155	3.37 (2.88–3.95)	3.34 (255.07)	3.34 (2.85)	1.74 (1.49)
Product quality issue	110	3.71 (3.08–4.48)	3.69 (215.95)	3.69 (3.06)	1.88 (1.57)
Eye swelling*	102	14.21 (11.69–17.27)	14.09 (1239.09)	14.07 (11.57)	3.81 (3.36)
Foreign body sensation in eyes*	102	2.63 (2.17–3.2)	2.62 (102.52)	2.62 (2.16)	1.39 (1.08)
Lacrimation increased*	97	78.36 (64.1–95.8)	77.73 (7264.85)	76.86 (62.87)	6.26 (5.14)
Eye pruritus*	87	15.4 (12.47–19.02)	15.29 (1160.2)	15.26 (12.36)	3.93 (3.41)
Visual impairment*	84	14.05 (11.33–17.42)	13.96 (1008.98)	13.93 (11.24)	3.8 (3.28)
Drug abuse	82	3.31 (2.66–4.11)	3.29 (131.07)	3.29 (2.65)	1.72 (1.36)
Intraocular pressure increased*	76	4.51 (3.6–5.65)	4.49 (206.16)	4.49 (3.58)	2.17 (1.77)
Dry eye*	76	30.27 (24.14–37.94)	30.08 (2127.56)	29.95 (23.89)	4.9 (4.11)
Therapeutic product effect decreased	74	8.51 (6.77–10.7)	8.46 (486.7)	8.45 (6.72)	3.08 (2.61)
Product prescribing error	70	3.49 (2.76–4.41)	3.47 (123.43)	3.47 (2.74)	1.8 (1.4)
Product substitution issue	66	7.3 (5.73–9.3)	7.26 (356.41)	7.26 (5.7)	2.86 (2.38)
Eye discharge*	65	5.42 (4.24–6.91)	5.39 (232.54)	5.39 (4.22)	2.43 (1.98)
Drug ineffective for unapproved indication	55	28.39 (21.77–37.03)	28.27 (1440.95)	28.16 (21.59)	4.82 (3.86)
Withdrawal syndrome	55	4.92 (3.77–6.41)	4.9 (170.76)	4.9 (3.76)	2.29 (1.81)
Product physical consistency issue	50	5.97 (4.52–7.89)	5.95 (205.96)	5.95 (4.5)	2.57 (2.03)
Eye disorder*	49	46.79 (35.31–62)	46.6 (2171.63)	46.29 (34.93)	5.53 (4.19)
Abnormal behavior	47	23.57 (17.69–31.4)	23.48 (1008.08)	23.4 (17.56)	4.55 (3.58)
Photophobia	46	7.3 (5.46–9.75)	7.27 (248.72)	7.27 (5.44)	2.86 (2.26)
Intentional overdose	44	5.88 (4.37–7.9)	5.86 (177.29)	5.86 (4.35)	2.55 (1.97)
Tinnitus	44	3.21 (2.38–4.31)	3.2 (66.53)	3.2 (2.38)	1.68 (1.18)
Product physical issue	43	12.23 (9.06–16.5)	12.19 (440.87)	12.17 (9.02)	3.6 (2.84)
Amnesia	43	3.4 (2.52–4.59)	3.39 (72.54)	3.39 (2.51)	1.76 (1.25)
Eye inflammation*	42	4.72 (3.49–6.4)	4.71 (122.75)	4.71 (3.48)	2.23 (1.67)
Aggression	42	9.62 (7.1–13.02)	9.59 (322.7)	9.57 (7.07)	3.26 (2.56)
Nightmare	41	3.3 (2.43–4.48)	3.29 (65.36)	3.29 (2.42)	1.72 (1.19)
Depressed level of consciousness	38	21.81 (15.86–30.01)	21.75 (749.9)	21.68 (15.76)	4.44 (3.36)
Nervousness	36	3.87 (2.79–5.37)	3.87 (76.48)	3.86 (2.79)	1.95 (1.37)
Disturbance in attention	36	5.32 (3.83–7.38)	5.3 (125.7)	5.3 (3.82)	2.41 (1.77)
Eyelid oedema*	33	4.76 (3.38–6.7)	4.75 (97.69)	4.75 (3.37)	2.25 (1.6)
Glaucoma*	31	3.02 (2.12–4.29)	3.01 (41.66)	3.01 (2.12)	1.59 (0.99)
Somnambulism*	31	2.95 (2.07–4.19)	2.94 (39.75)	2.94 (2.07)	1.56 (0.96)
Product residue present	30	12.84 (8.97–18.37)	12.81 (326.01)	12.79 (8.93)	3.68 (2.69)
Psychotic disorder	29	7.75 (5.38–11.16)	7.73 (169.86)	7.73 (5.36)	2.95 (2.13)
Panic attack	28	19.87 (13.71–28.81)	19.83 (499.15)	19.77 (13.64)	4.31 (3.05)
Ocular discomfort*	27	10.56 (7.24–15.42)	10.54 (232.91)	10.53 (7.21)	3.4 (2.43)
Product delivery mechanism issue	27	4.96 (3.4–7.24)	4.95 (85.16)	4.95 (3.39)	2.31 (1.57)
Poisoning	26	3.74 (2.54–5.49)	3.73 (52.03)	3.73 (2.54)	1.9 (1.2)
Restlessness	26	14.59 (9.93–21.45)	14.56 (327.8)	14.54 (9.89)	3.86 (2.72)
Extra dose administered	26	30.98 (21.07–45.56)	30.92 (749.33)	30.78 (20.93)	4.94 (3.31)
Dysarthria*	26	7.34 (4.99–10.79)	7.33 (141.92)	7.32 (4.98)	2.87 (2.01)

Abbreviations: CI, confidence interval; EBGM, empirical Bayesian geometric mean; EBGM05, the lower limit of the 95% CI of EBGM; IC, information component; IC025, the lower limit of the 95% CI of the IC; PRR, proportional reporting ratio; PT, preferred term; ROR, reporting odds ratio.

* For adverse events not documented in the instructions.

### 3.3 Subgroup Analyses

Subgroup analyses were subsequently performed on the 50 highest‐frequency adverse events (Tables S4 and S5). Gender‐specific signals revealed that amnesia, depressed level of consciousness, disturbance in attention, psychotic disorder, and thinking abnormal were exclusive to males, whereas eye infection, conjunctivitis, eye allergy, corneal disorder, blepharitis, and keratitis were unique to females. Age‐subgroup analysis indicated that pediatric patients required additional monitoring for seizure and hypertensive crisis; adults warranted attention to cardio‐respiratory arrest and dysarthria; and visual impairment and tinnitus should be vigilantly monitored in elderly patients. Details were provided in Tables S6, S7, and S8.

### 3.4 Time to Onset and Weibull Distribution Analysis of Adverse Events

**FIGURE 3:**
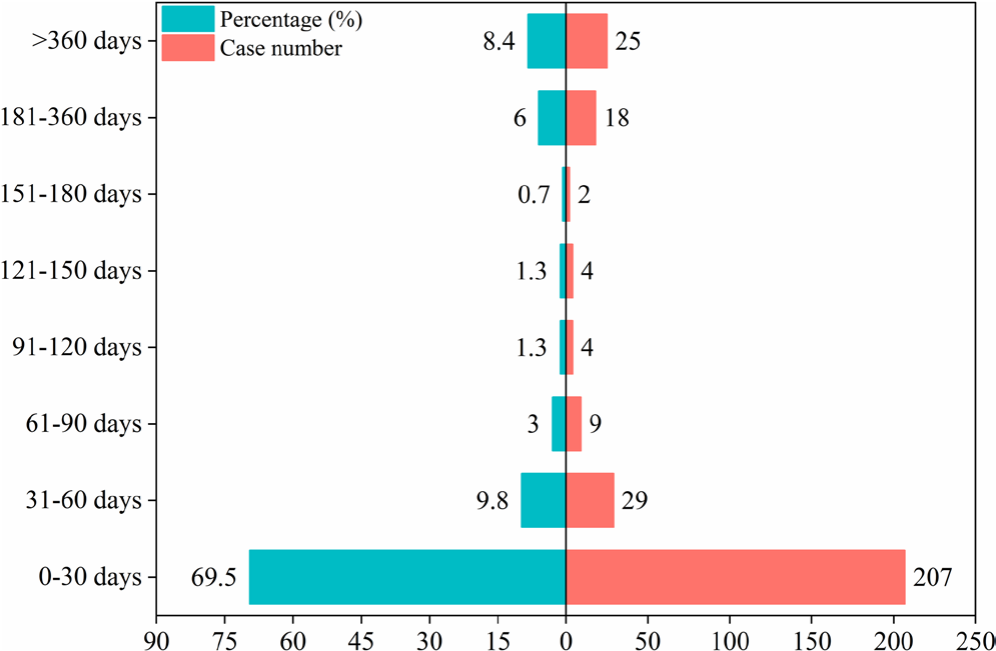
Time to onset of temazepam‐associated adverse events.

**FIGURE 4:**
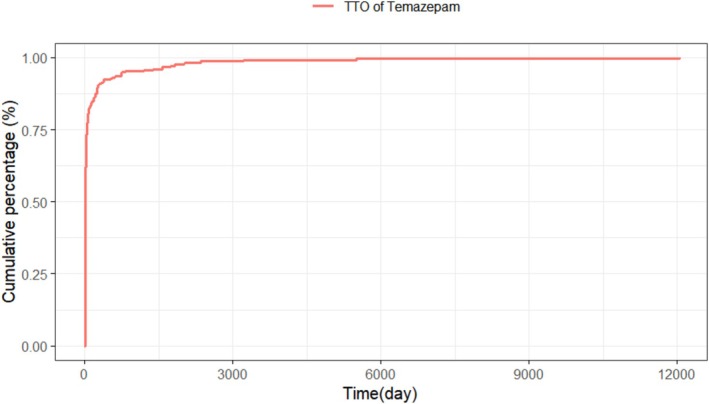
Cumulative incidence of temazepam‐associated adverse events over time. TTO, time to onset.

Data on the onset times of adverse events were available for 298 patients. As shown in Figure 3, the majority of temazepam‐related adverse events manifested within the first 30 days (69.5%) after administration, followed by days 31 to 60 (9.8%). Notably, 14.4% of events emerged after 6 months, underscoring potential long‐term risks. The median time to onset was 8 days. The cumulative incidence curve for temazepam‐related adverse events was presented in Figure 4. The Weibull distribution was used to model onset times (Table 4); the results indicated an early‐failure pattern with a decline in temazepam‐related adverse events over time.

**TABLE 4:** Time to onset of temazepam‐associated adverse events and Weibull distribution analysis.

Drug	TTO (days)		Weibull distribution		
	Case reports	Median (d) (IQR)	Scale parameter: α (95% CI)	Shape parameter: β (95% CI)	Type
Temazepam	298	8 (43)	38.05 (28.04, 52.41)	0.41 (0.37, 0.44)	Early failure

Abbreviations: CI, confidence interval; IQR, interquartile range; TTO, time to onset.

## 4 Discussion

By mining the large‐scale FAERS database, we performed a real‐world pharmacovigilance assessment of temazepam‐related adverse events. The results indicate that the proportion of temazepam‐related adverse event reports from female patients was substantially higher than from male patients, consistent with the gender‐specific epidemiology of insomnia [18]. Patients aged 18 to 64 years accounted for the largest group, followed by those aged 65 to 85 years, reflecting broader temazepam use in these age groups. United States reports predominated, with Britain second, consistent with temazepam being FDA‐approved and first marketed in the United States and with Britain being one of the primary locations for the drug's early clinical development [19, 20]. The main sources of reports were consumers and healthcare professionals, demonstrating that several stakeholders were actively engaged in the pharmacovigilance system. Report numbers increased steadily from 2004 to 2019 and then declined gradually, possibly owing to the introduction of competitive medications, the stricter enforcement of clinical sedative drug control, a more standard clinical application prompted by experience, and reporting weariness.

Temazepam, a benzodiazepine hypnotic, mainly resulted in adverse events in systems involving general disorders and administration site conditions, eye disorders, injury, poisoning, and procedural complications, psychiatric disorders, and nervous system disorders. That was indicative of the drug's extensive spectrum of impact on organ systems. Drug ineffective, eye irritation, toxicity to various agents, vision blurred, and completed suicide were the most frequently reported adverse events. The significance of cautious and monitored medication administration is highlighted by these findings. Notably, although the drug label mentioned that the risk of vision blurred, no other ocular risks were included in the labeling. Our current study, however, identified a large number of additional adverse events related to the ocular system, including eye irritation, eye pain, ocular hyperaemia, eye swelling, visual impairment, intraocular pressure increased, photophobia, eye inflammation, and glaucoma. Certain of these risk signals showed a relatively high frequency of occurrence, which calls for additional clinical attention.

Additionally, considering the potential for benzodiazepine hypnotic abuse, we identified events such as drug abuse, intentional overdose, product residue present, poisoning, and extra dose administered, which likely reflect patient misuse. These phenomena are well documented [21, 22]. Benzodiazepines remain among the most abused prescription drugs worldwide [23]. By positively modulating gamma aminobutyric acid (GABA) receptors in neighboring interneurons, benzodiazepines boost the firing of dopamine neurons in the ventral tegmental region, cause mesolimbic dopamine release, and intensify dopamine's rewarding and euphoric effects [24]. Previous studies also showed that concurrent use of opioids, nicotine, or alcohol amplifies benzodiazepine effects, further increasing overdose and addiction risk [25, 26, 27]. Interestingly, the most frequently reported adverse event in our study was drug ineffective; this finding dovetails with the concurrently observed risk signals of potential abuse. Drug ineffective might induce patients to escalate their dosage or extend the course of treatment without supervision by physicians, strengthening the risk of reliance and abuse. It is important to note that this signal is intrinsically vulnerable to indication bias in spontaneous reports for a subjective symptom such as insomnia, an inherent limitation of pharmacovigilance databases. Actually, instead of being the direct result of medication administration, the reported ineffectiveness of the drug was more likely to be ascribed to inadequate control of the preexisting insomnia disease. Consistently, patients' reports of drug ineffective have a significantly greater ratio than those reported from healthcare professionals due to indication bias [28]. Additionally, drug ineffective might indirectly reflect the patients' tolerance development. This signal may not be exclusive to temazepam, but rather a dynamic change experienced while treating insomnia that has to be taken seriously. These results underline the necessity of rigorous clinical monitoring of patient compliance and dose self‐adjustment, as well as the pressing need for more thorough, evidence‐based regulation, intervention, and preventative initiatives. Sustained endeavors to undertake research and develop targeted approaches to effectively address drug abuse must be maintained, as evidenced by the increasing number of individuals seeking assistance with benzodiazepine reliance and withdrawal symptoms. The present study has the potential to inform subsequent contexts with the aim of curbing drug abuse.

We identified several behavioral risk signals, including abnormal behavior, aggression, amnesia, and depressed level of consciousness. Previous studies showed that benzodiazepine exposure is linked to deterioration in cognitive and reactive capacity [29]. Multiple studies have reported that benzodiazepine use increases the risk of motor‐vehicle accidents, psychomotor impairment, and cognitive dysfunction such as anterograde amnesia, leading to impaired short‐term memory and increased forgetfulness [30, 31]. It might be because benzodiazepines impair episodic implicit memory through their toxic effects on the central nervous system. They also caused disinhibition, which makes it harder for the user to recognize dangerous behaviors or actions [32]. 1%–20% of patients exhibited aggression or hostility following benzodiazepine treatment [33]. These risks were frequently linked to an increase in concerning violent behavior, even criminal activity [34]. Given the seriousness of these potential risk signals and the results of our study, caution should be taken in clinical practice, and patients should be advised of any adverse events when taking benzodiazepines for insomnia, including potentially adverse effects on driving or other activities that demand a high level of attention during the period after therapy. Additionally, physicians should carefully evaluate patients' mental health both before and throughout therapy, adjusting their approaches as necessary.

Interestingly, our research uncovered some parasomnias‐related adverse effects, including nightmare and somnambulism, that have been under‐reported in the literature. These findings suggested temazepam may precipitate parasomnia symptoms during sleep promotion, warranting increased clinical vigilance. The exact mechanisms are unclear, but they may relate to the way temazepam affected the transitions between the stages of sleep, involving both rapid eye movement (REM) sleep and non‐rapid eye movement (NREM) sleep, as well as wakefulness. Temazepam, prescribed for sleep disorders, has been shown to adjust the structure of sleep through lengthening NREM and decreasing REM sleep [35]. These changes in stages of sleep usually pertain to specific patterns of oscillatory activity in the brain, which could result in the boundaries between these stages becoming blurred and dissociated sleep phenomena emerging [36, 37]. In specific, the insufficient separation of wakefulness, REM sleep, and NREM sleep might cause recurrent cortical arousals and change sleep inertia, which could result in parasomnias. Importantly, the signal strength in the FAERS database represents the reported relative frequency rather than causation. Further confirmatory studies (e.g., cohort studies) are needed to clarify the underlying mechanisms of temazepam's association with parasomnias. Additionally, such effects might be made worse by several co‐prescribed drugs and concomitant diseases in some situations. Implicated drugs included tricyclic antidepressants, bupropion, selective serotonin reuptake inhibitors, lithium, second‐generation antipsychotics, and fluoroquinolone antimicrobials, while clinical risk factors included migraine attacks, febrile episodes, vitiligo, and thyrotoxicosis [38, 39, 40]. Among patients with sleep problems, these parasomnia episodes were substantially linked to elevated distress levels. However, these adverse events were frequently underreported. A study revealed that nearly one in four individuals with elevated psychopathology scores experienced clinically significant parasomnia phenomena, while over 60% had never disclosed these events to a healthcare professional [41]. Given their potential for interfering with sleep, bringing risks for injury, and serving as indicators of sleep instability, the clinical significance of adverse events related to parasomnias shouldn't be undervalued. Physicians prescribing temazepam are advised to maintain close monitoring of each patient's individual reactions and sleep patterns, especially during dose adjustments. Strategies including timing modifications, dose reduction, or switching to alternative agents may be used to mitigate these undesirable effects when necessary.

Notably, our study also identified a significant amount of temazepam‐related ocular toxicities reaction, including eye irritation, vision blurred, eye pain, ocular hyperaemia, dry eye, eye swelling, visual impairment, lacrimation increased, intraocular pressure increased, glaucoma, eye inflammation, photophobia, none of which were listed in the existing drug label except for vision blurred. Higher reporting frequency was shown for some of these adverse events. Temazepam, as one of the most commonly prescribed drugs in benzodiazepines, enhances the effects of gamma aminobutyric acid (GABA) to produce sedative, hypnotic, and muscle‐relaxing effects [42]. Because of this function, benzodiazepines were implicated in influencing the pupillary sphincter and determining the iridocorneal angle narrowing. Ocular function regulation was intimately linked to the iridocorneal angle and sphincter pupillae, and their lesions might impair ocular regulation [43]. Specifically, temazepam potentiates GABA receptors on preganglionic sympathetic terminals within the ciliary body, which decreases aqueous outflow facility and elevates intraocular pressure; concurrent GABA‐mediated inhibition of the iris dilator allows posterior iris bowing, mechanically narrowing the iridocorneal angle and predisposing to glaucoma [44, 45]. Early studies have confirmed that individuals might develop a variety of ocular side effects during the therapy of benzodiazepines, such as eye inflammation (e.g., allergic conjunctivitis), eye irritation, vision blurred, and blepharospasm [46, 47, 48]. Furthermore, it has been established that using benzodiazepines increases the potential of developing glaucoma. Glaucoma, one of the most common causes of blindness worldwide, is a chronically proceeding neurodegenerative disorder of the optic nerve [44]. Intraocular pressure increase is a major risk factor for glaucoma development [49]. Eye pain, ocular hyperaemia, eye swelling, visual impairment, lacrimation increased, and photophobia could all be early symptoms of glaucoma [50]; these manifestations align with overstimulation of GABA receptors on lacrimal acinar cells and inhibitory interneurons in the pontine lacrimal nucleus, producing the observed ocular risk signals, including dry eye, irritation and reflex lacrimation [43]. Park et al. [51] found that glaucoma occurrence was significantly associated with benzodiazepine use; the risk was highest among first‐time users treated within 7 days, with no difference between short acting and long acting agents. Kim et al. observed a similar link in 6709 patients (aOR 1.40; 95% CI 1.27–1.54) for both short half‐life (aOR 1.40; 95% CI 1.24–1.57) and long half‐life (aOR 1.33; 95% CI 1.18–1.50) benzodiazepines [52]. Drug‐induced glaucoma is an ophthalmic emergency that can cause irreversible visual loss if not promptly diagnosed and treated [53]. Although the signal strength detected in our pharmacovigilance study reflects statistical associations rather than causality, the large number of temazepam‐related ophthalmological risk signals uncovered in our research sounds an unequivocal clinical alarm. These findings reflected the wide‐ranging effects of temazepam on the ocular system. Therefore, we proposed that any patient with even one risk factor for glaucoma, such as age over 60 years, hyperopia, family history of glaucoma, should receive baseline intraocular pressure measuremen before the first temazepam. We additionally recommended routine intraocular pressure monitoring during treatment.

Subgroup analyses showed that male patients required additional monitoring for amnesia, depressed level of consciousness, disturbance in attention, psychotic disorder, and thinking abnormality, whereas female patients benefited from regular assessment for eye infection, conjunctivitis, eye allergy, corneal disorder, blepharitis, and keratitis. Exploring sex distinctions in drug use results is important, given that certain biological distinctions between males and females can affect both the short‐ and long‐term effects of drugs taken by gender. Multifactorial interactions may be the cause of the observed gender difference in the occurrence of adverse events. Previous research has indicated that hormone levels, drug metabolism, drug responsiveness, body fat distribution, and drug distribution volume vary by sex [54, 55, 56]. These variations should be considered in therapeutic settings, even if the exact mechanisms by which sexual dimorphism affects temazepam adverse reactions are yet established. Additionally, disparities in adverse events were noted among age groups. More focus should be placed on the temazepam reaction to seizure and hypertensive crisis in pediatric patients. Adult patients should be closely monitored for the risks of amnesia, cardio‐respiratory arrest, and dysarthria, since these conditions can negatively impact their quality of life. Vigilance about visual impairment and tinnitus was needed in elderly individuals.

Analysis of adverse‐event onset provided important safety information for temazepam. Notably, 69.4% of reactions arose within the first 30 days of treatment, highlighting the importance of early monitoring to maximize therapeutic benefit. Adverse event frequency declined thereafter, consistent with our Weibull model which showed early‐failure characteristics and a progressive reduction in risk over time. Furthermore, 14.5% of events occurred more than 6 months after initiation. This might be attributed to delayed toxicity or the long‐term therapeutic impact, with some people reporting adverse events later because of individual variations [57]. These findings emphasized the need for long‐term follow‐up to capture rare but clinically relevant events.

Despite providing clinically significant insights into temazepam‐associated adverse events, this study had several inherent limitations. First, the FAERS relies on spontaneous reporting, which is vulnerable to incomplete information (e.g., insufficient patient demographics and dosage details), under‐reporting (e.g., healthcare providers or patients may not identify certain signals as adverse events or fail to report them), and a lack of denominator data, precluding the calculation of true incidence rates. Second, the majority of reports originate from the United States and Europe, while other regions such as Africa and several Asian countries remain underrepresented, potentially limiting generalizability. For example, differences in patient demographics, prescription practices, and healthcare systems between these regions may affect the types and frequencies of reported adverse events. Third, confounding factors, including baseline patient characteristics, comorbidities, and genetic factors that may influence adverse event incidence, were not accounted for in this investigation due to database constraints. However, our statistical results revealed only a potential correlation between temazepam and certain adverse events, not a causal relationship. Future studies assessing temazepam's safety profile should consider employing more rigorous prospective designs that integrate clinical trial data with real‐world epidemiological evidence to enable more robust risk assessment.

## 5 Conclusion

A comprehensive pharmacovigilance analysis of temazepam‐related adverse events was conducted using real‐world data from the large‐scale FAERS database. The most frequently reported events were drug inefficacy, toxicity to various agents, eye irritation, blurred vision, and eye pain. Several ocular risk signals absent from the product label were identified, along with behavioral risk signals including abnormal behavior, aggression, amnesia, and depressed level of consciousness, and parasomnia‐related events such as nightmare and somnambulism. These findings underscored the need for enhanced monitoring, particularly within the first 30 days of treatment, to facilitate prompt management of temazepam‐associated adverse events. It is recommended that doctors and patients regularly assess the potential risks connected to its use, intervening and amending therapy regimens as necessary.

## Author Contributions

Zujun Wen, Xiang Liu, and Peng Liu designed the research. Zujun Wen and Xiang Liu collected, analyzed the data, and drafted the manuscript. Zujun Wen, Xiang Liu, and Peng Liu revised the manuscript. All authors contributed to the article and approved the submitted version.

## Funding

The authors have nothing to report.

## Ethics Statement

This study was based on publicly available, de‐identified data from the FAERS database. No personally identifiable information or patient interventions were involved. Therefore, formal ethical committee approval and informed consent were not required under applicable guidelines and regulations.

## Conflicts of Interest

The authors declare no conflicts of interest.

## Supporting information


**Table S1:** Fourfold table of disproportionality analysis.
**Table S2:** Formula and criteria of four algorithms for adverse event signal detection.
**Table S3:** All adverse events meeting the positive signal threshold at the preferred term (PT) level from FAERS data.
**Table S4:** Top 50 most frequent adverse events for temazepam at the preferred term (PT) level in males from FAERS data.
**Table S5:** Top 50 most frequent adverse events for temazepam at the preferred term (PT) level in females from FAERS data.
**Table S6:** Adverse events at the preferred term (PT) level for temazepam in patients aged under 18 from FAERS data.
**Table S7:** Adverse events at the preferred term (PT) level for temazepam in patients aged 18 to 65 from FAERS data.
**Table S8:** Adverse events at the preferred term (PT) level for temazepam in patients aged over 65 from FAERS data.

## Data Availability

The data that support the findings of this study are openly available in FDA Adverse Event Reporting System at https://www.fda.gov.
