# Supplementary material for: The Real‐World Safety Profile of Temazepam: A 20‐Year Pharmacovigilance Analysis Based on the Large‐Scale FAERS Database
Source: CNS Neurosci Ther. 2026 Mar 23;32(3):e70836. doi: 10.1002/cns.70836 (PMC13093608; doi:10.1002/cns.70836)
Supplement: Supplementary file 1 — Table S1: Fourfold table of disproportionality analysis. Table S2: Formula and criteria of four algorithms for adverse event signal detection. Table S3: All adverse events meeting the positive signal threshold at the preferred term (PT) level from FAERS data. Table S4: Top 50 most frequent adverse events for temazepam at the preferred term (PT) level in males from FAERS data. Table S5: Top 50 most frequent adverse events for temazepam at the preferred term (PT) level in females from FAERS data. Table S6: Adverse events at the preferred term (PT) level for temazepam in patients aged under 18 from FAERS data. Table S7: Adverse events at the preferred term (PT) level for temazepam in patients aged 18 to 65 from FAERS data. Table S8: Adverse events at the preferred term (PT) level for temazepam in patients aged over 65 from FAERS data. [file CNS-32-e70836-s001.docx]

**Supplementary Table S1**. Fourfold table of disproportionality analysis.

|  | Reports with target adverse event | Reports with other adverse events |
| --- | --- | --- |
| Reports as the primary suspected drug | a | b |
| Reports with other drugs as the primary suspected drug | c | d |

Abbreviation: a, number of reports containing both temazepam and target adverse event; b, number of reports containing other adverse events of temazepam; c, number of reports containing target adverse event of other drugs; d, number of reports containing other drugs and other adverse events

**Supplementary Table S2**. Formula and criteria of four algorithms for adverse event signal detection.

| Algorithm | Formula | Criteria |
| --- | --- | --- |
| ROR | ROR = $\frac{a/c}{b/d}$  95% CI = $e^{\ln(ROR)\pm1.96\sqrt{\frac{1}{a}+\frac{1}{b}+\frac{1}{c}+\frac{1}{d}}}$ | N ≥ 3,  Lower limit of 95% CI > 1 |
| PRR | PRR = $\frac{a}{a+b}/\frac{c}{c+d}$  χ^2^ = $\frac{[(ad-bc)^2](a+b+c+d)}{[(a+b)(c+d)(a+c)(b+d)}$ | PRR≥2, χ^2^≥4, N≥3 |
| BCPNN | IC = log_2_$\frac{a(a+b+c+d)}{(a+c)(a+b)}$  IC025 = $e^{\ln(IC)-1.96\sqrt{\frac{1}{a}+\frac{1}{b}+\frac{1}{c}+\frac{1}{d}}}$ | IC025 > 0 |
| MGPS | EBGM = $\frac{a(a+b+c+d)}{(a+c)(a+b)}$  EBGM05 = $e^{\ln(EBGM)-1.96\sqrt{\frac{1}{a}+\frac{1}{b}+\frac{1}{c}+\frac{1}{d}}}$ | EBGM05>2 |

Abbreviation: N, number of reports; ROR, reporting odds ratio; CI, confidence interval; PRR, proportional reporting ratio; χ2, chi-squared; IC, information component; IC025, the lower limit of 95%CI of the IC; EBGM, empirical bayesian geometric mean; EBGM05, lower limit of 95% confidence interval of EBGM.

**Supplementary Table S3**. All adverse events meeting the positive signal threshold at the PT level from FAERS data.

| **PT** | **Case numbers** | **ROR(95%CI)** | **PRR(χ^2^)** | **EBGM(EBGM05)** | **IC(IC025)** |
| --- | --- | --- | --- | --- | --- |
| Drug ineffective* | 668 | 2.63 ( 2.43 - 2.84 ) | 2.54 ( 634.79 ) | 2.53 ( 2.34 ) | 1.34 ( 1.22 ) |
| Eye irritation* | 263 | 26.1 ( 23.09 - 29.5 ) | 25.55 ( 6185.42 ) | 25.46 ( 22.52 ) | 4.67 ( 4.36 ) |
| Eye pain* | 230 | 22.59 ( 19.82 - 25.74 ) | 22.17 ( 4639.16 ) | 22.1 ( 19.4 ) | 4.47 ( 4.15 ) |
| Toxicity to various agents | 224 | 6.19 ( 5.42 - 7.06 ) | 6.09 ( 954.83 ) | 6.08 ( 5.33 ) | 2.61 ( 2.38 ) |
| Vision blurred | 221 | 8.63 ( 7.55 - 9.86 ) | 8.49 ( 1461.18 ) | 8.48 ( 7.42 ) | 3.08 ( 2.84 ) |
| Insomnia* | 213 | 4.12 ( 3.6 - 4.72 ) | 4.07 ( 494.77 ) | 4.07 ( 3.55 ) | 2.02 ( 1.8 ) |
| Completed suicide | 208 | 13.64 ( 11.89 - 15.65 ) | 13.42 ( 2389.4 ) | 13.4 ( 11.68 ) | 3.74 ( 3.46 ) |
| Ocular hyperaemia* | 155 | 17.75 ( 15.15 - 20.81 ) | 17.54 ( 2412.57 ) | 17.49 ( 14.93 ) | 4.13 ( 3.75 ) |
| Product use in unapproved indication | 155 | 3.37 ( 2.88 - 3.95 ) | 3.34 ( 255.07 ) | 3.34 ( 2.85 ) | 1.74 ( 1.49 ) |
| Product quality issue | 110 | 3.71 ( 3.08 - 4.48 ) | 3.69 ( 215.95 ) | 3.69 ( 3.06 ) | 1.88 ( 1.57 ) |
| Eye swelling* | 102 | 14.21 ( 11.69 - 17.27 ) | 14.09 ( 1239.09 ) | 14.07 ( 11.57 ) | 3.81 ( 3.36 ) |
| Foreign body sensation in eyes* | 102 | 2.63 ( 2.17 - 3.2 ) | 2.62 ( 102.52 ) | 2.62 ( 2.16 ) | 1.39 ( 1.08 ) |
| Lacrimation increased* | 97 | 78.36 ( 64.1 - 95.8 ) | 77.73 ( 7264.85 ) | 76.86 ( 62.87 ) | 6.26 ( 5.14 ) |
| Eye pruritus* | 87 | 15.4 ( 12.47 - 19.02 ) | 15.29 ( 1160.2 ) | 15.26 ( 12.36 ) | 3.93 ( 3.41 ) |
| Visual impairment* | 84 | 14.05 ( 11.33 - 17.42 ) | 13.96 ( 1008.98 ) | 13.93 ( 11.24 ) | 3.8 ( 3.28 ) |
| Drug abuse | 82 | 3.31 ( 2.66 - 4.11 ) | 3.29 ( 131.07 ) | 3.29 ( 2.65 ) | 1.72 ( 1.36 ) |
| Intraocular pressure increased* | 76 | 4.51 ( 3.6 - 5.65 ) | 4.49 ( 206.16 ) | 4.49 ( 3.58 ) | 2.17 ( 1.77 ) |
| Dry eye* | 76 | 30.27 ( 24.14 - 37.94 ) | 30.08 ( 2127.56 ) | 29.95 ( 23.89 ) | 4.9 ( 4.11 ) |
| Therapeutic product effect decreased | 74 | 8.51 ( 6.77 - 10.7 ) | 8.46 ( 486.7 ) | 8.45 ( 6.72 ) | 3.08 ( 2.61 ) |
| Product prescribing error | 70 | 3.49 ( 2.76 - 4.41 ) | 3.47 ( 123.43 ) | 3.47 ( 2.74 ) | 1.8 ( 1.4 ) |
| Product substitution issue | 66 | 7.3 ( 5.73 - 9.3 ) | 7.26 ( 356.41 ) | 7.26 ( 5.7 ) | 2.86 ( 2.38 ) |
| Eye discharge* | 65 | 5.42 ( 4.24 - 6.91 ) | 5.39 ( 232.54 ) | 5.39 ( 4.22 ) | 2.43 ( 1.98 ) |
| Drug ineffective for unapproved indication | 55 | 28.39 ( 21.77 - 37.03 ) | 28.27 ( 1440.95 ) | 28.16 ( 21.59 ) | 4.82 ( 3.86 ) |
| Withdrawal syndrome | 55 | 4.92 ( 3.77 - 6.41 ) | 4.9 ( 170.76 ) | 4.9 ( 3.76 ) | 2.29 ( 1.81 ) |
| Product physical consistency issue | 50 | 5.97 ( 4.52 - 7.89 ) | 5.95 ( 205.96 ) | 5.95 ( 4.5 ) | 2.57 ( 2.03 ) |
| Eye disorder* | 49 | 46.79 ( 35.31 - 62 ) | 46.6 ( 2171.63 ) | 46.29 ( 34.93 ) | 5.53 ( 4.19 ) |
| Abnormal behaviour | 47 | 23.57 ( 17.69 - 31.4 ) | 23.48 ( 1008.08 ) | 23.4 ( 17.56 ) | 4.55 ( 3.58 ) |
| Photophobia | 46 | 7.3 ( 5.46 - 9.75 ) | 7.27 ( 248.72 ) | 7.27 ( 5.44 ) | 2.86 ( 2.26 ) |
| Intentional overdose | 44 | 5.88 ( 4.37 - 7.9 ) | 5.86 ( 177.29 ) | 5.86 ( 4.35 ) | 2.55 ( 1.97 ) |
| Tinnitus | 44 | 3.21 ( 2.38 - 4.31 ) | 3.2 ( 66.53 ) | 3.2 ( 2.38 ) | 1.68 ( 1.18 ) |
| Product physical issue | 43 | 12.23 ( 9.06 - 16.5 ) | 12.19 ( 440.87 ) | 12.17 ( 9.02 ) | 3.6 ( 2.84 ) |
| Amnesia | 43 | 3.4 ( 2.52 - 4.59 ) | 3.39 ( 72.54 ) | 3.39 ( 2.51 ) | 1.76 ( 1.25 ) |
| Eye inflammation* | 42 | 4.72 ( 3.49 - 6.4 ) | 4.71 ( 122.75 ) | 4.71 ( 3.48 ) | 2.23 ( 1.67 ) |
| Aggression | 42 | 9.62 ( 7.1 - 13.02 ) | 9.59 ( 322.7 ) | 9.57 ( 7.07 ) | 3.26 ( 2.56 ) |
| Nightmare | 41 | 3.3 ( 2.43 - 4.48 ) | 3.29 ( 65.36 ) | 3.29 ( 2.42 ) | 1.72 ( 1.19 ) |
| Depressed level of consciousness | 38 | 21.81 ( 15.86 - 30.01 ) | 21.75 ( 749.9 ) | 21.68 ( 15.76 ) | 4.44 ( 3.36 ) |
| Nervousness | 36 | 3.87 ( 2.79 - 5.37 ) | 3.87 ( 76.48 ) | 3.86 ( 2.79 ) | 1.95 ( 1.37 ) |
| Disturbance in attention | 36 | 5.32 ( 3.83 - 7.38 ) | 5.3 ( 125.7 ) | 5.3 ( 3.82 ) | 2.41 ( 1.77 ) |
| Eyelid oedema* | 33 | 4.76 ( 3.38 - 6.7 ) | 4.75 ( 97.69 ) | 4.75 ( 3.37 ) | 2.25 ( 1.6 ) |
| Glaucoma* | 31 | 3.02 ( 2.12 - 4.29 ) | 3.01 ( 41.66 ) | 3.01 ( 2.12 ) | 1.59 ( 0.99 ) |
| Somnambulism* | 31 | 2.95 ( 2.07 - 4.19 ) | 2.94 ( 39.75 ) | 2.94 ( 2.07 ) | 1.56 ( 0.96 ) |
| Product residue present | 30 | 12.84 ( 8.97 - 18.37 ) | 12.81 ( 326.01 ) | 12.79 ( 8.93 ) | 3.68 ( 2.69 ) |
| Psychotic disorder | 29 | 7.75 ( 5.38 - 11.16 ) | 7.73 ( 169.86 ) | 7.73 ( 5.36 ) | 2.95 ( 2.13 ) |
| Panic attack | 28 | 19.87 ( 13.71 - 28.81 ) | 19.83 ( 499.15 ) | 19.77 ( 13.64 ) | 4.31 ( 3.05 ) |
| Ocular discomfort* | 27 | 10.56 ( 7.24 - 15.42 ) | 10.54 ( 232.91 ) | 10.53 ( 7.21 ) | 3.4 ( 2.43 ) |
| Product delivery mechanism issue | 27 | 4.96 ( 3.4 - 7.24 ) | 4.95 ( 85.16 ) | 4.95 ( 3.39 ) | 2.31 ( 1.57 ) |
| Poisoning | 26 | 3.74 ( 2.54 - 5.49 ) | 3.73 ( 52.03 ) | 3.73 ( 2.54 ) | 1.9 ( 1.2 ) |
| Restlessness | 26 | 14.59 ( 9.93 - 21.45 ) | 14.56 ( 327.8 ) | 14.54 ( 9.89 ) | 3.86 ( 2.72 ) |
| Extra dose administered | 26 | 30.98 ( 21.07 - 45.56 ) | 30.92 ( 749.33 ) | 30.78 ( 20.93 ) | 4.94 ( 3.31 ) |
| Dysarthria* | 26 | 7.34 ( 4.99 - 10.79 ) | 7.33 ( 141.92 ) | 7.32 ( 4.98 ) | 2.87 ( 2.01 ) |
| Cardio-respiratory arrest | 25 | 3.56 ( 2.41 - 5.28 ) | 3.56 ( 46.02 ) | 3.56 ( 2.4 ) | 1.83 ( 1.13 ) |
| Product dispensing error | 25 | 3.32 ( 2.25 - 4.92 ) | 3.32 ( 40.53 ) | 3.32 ( 2.24 ) | 1.73 ( 1.04 ) |
| Product container issue | 24 | 3.39 ( 2.27 - 5.07 ) | 3.39 ( 40.44 ) | 3.39 ( 2.27 ) | 1.76 ( 1.05 ) |
| Product availability issue | 24 | 3.07 ( 2.06 - 4.59 ) | 3.07 ( 33.45 ) | 3.07 ( 2.05 ) | 1.62 ( 0.92 ) |
| Somnolence | 23 | 10.11 ( 6.72 - 15.23 ) | 10.1 ( 188.24 ) | 10.08 ( 6.7 ) | 3.33 ( 2.28 ) |
| Burning sensation | 23 | 3.1 ( 2.06 - 4.66 ) | 3.09 ( 32.57 ) | 3.09 ( 2.05 ) | 1.63 ( 0.92 ) |
| Conjunctivitis | 22 | 6.37 ( 4.19 - 9.68 ) | 6.36 ( 99.39 ) | 6.36 ( 4.18 ) | 2.67 ( 1.76 ) |
| Abnormal dreams | 22 | 4.21 ( 2.77 - 6.4 ) | 4.2 ( 53.73 ) | 4.2 ( 2.77 ) | 2.07 ( 1.28 ) |
| Abnormal sensation in eye | 22 | 28.53 ( 18.76 - 43.39 ) | 28.48 ( 581.02 ) | 28.37 ( 18.66 ) | 4.83 ( 3.09 ) |
| Respiratory depression | 21 | 9.39 ( 6.12 - 14.41 ) | 9.37 ( 156.85 ) | 9.36 ( 6.1 ) | 3.23 ( 2.15 ) |
| Instillation site pain | 21 | 35.43 ( 23.07 - 54.42 ) | 35.37 ( 697.8 ) | 35.19 ( 22.91 ) | 5.14 ( 3.17 ) |
| Product packaging quantity issue | 21 | 10.66 ( 6.94 - 16.35 ) | 10.64 ( 183.13 ) | 10.62 ( 6.92 ) | 3.41 ( 2.27 ) |
| Instillation site erythema | 20 | 175.41 ( 112.49 - 273.52 ) | 175.12 ( 3375.56 ) | 170.75 ( 109.5 ) | 7.42 ( 3.59 ) |
| Asthenopia | 18 | 16.57 ( 10.43 - 26.33 ) | 16.55 ( 262.33 ) | 16.51 ( 10.39 ) | 4.05 ( 2.52 ) |
| Instillation site irritation | 18 | 103.71 ( 65.09 - 165.25 ) | 103.56 ( 1800.83 ) | 102.02 ( 64.03 ) | 6.67 ( 3.35 ) |
| Eyelids pruritus | 17 | 30.44 ( 18.9 - 49.03 ) | 30.4 ( 481.18 ) | 30.27 ( 18.79 ) | 4.92 ( 2.84 ) |
| Swelling of eyelid | 16 | 15.76 ( 9.64 - 25.74 ) | 15.74 ( 220.29 ) | 15.7 ( 9.61 ) | 3.97 ( 2.37 ) |
| Thinking abnormal | 16 | 4.45 ( 2.72 - 7.27 ) | 4.45 ( 42.71 ) | 4.44 ( 2.72 ) | 2.15 ( 1.18 ) |
| Eyelid margin crusting | 16 | 31.69 ( 19.39 - 51.8 ) | 31.65 ( 472.71 ) | 31.51 ( 19.27 ) | 4.98 ( 2.79 ) |
| Hangover | 15 | 20.74 ( 12.49 - 34.45 ) | 20.72 ( 280.68 ) | 20.66 ( 12.44 ) | 4.37 ( 2.49 ) |
| Product prescribing issue | 15 | 4.88 ( 2.94 - 8.09 ) | 4.87 ( 46.12 ) | 4.87 ( 2.93 ) | 2.28 ( 1.25 ) |
| Middle insomnia | 14 | 4.06 ( 2.4 - 6.86 ) | 4.06 ( 32.23 ) | 4.05 ( 2.4 ) | 2.02 ( 1.01 ) |
| Erythema of eyelid | 14 | 13.96 ( 8.26 - 23.58 ) | 13.94 ( 167.83 ) | 13.91 ( 8.23 ) | 3.8 ( 2.16 ) |
| Product use complaint | 14 | 4.65 ( 2.75 - 7.85 ) | 4.64 ( 39.98 ) | 4.64 ( 2.75 ) | 2.21 ( 1.15 ) |
| Coordination abnormal | 14 | 5.5 ( 3.26 - 9.3 ) | 5.5 ( 51.5 ) | 5.5 ( 3.25 ) | 2.46 ( 1.33 ) |
| Therapeutic product effect increased | 13 | 14.23 ( 8.25 - 24.52 ) | 14.21 ( 159.36 ) | 14.18 ( 8.23 ) | 3.83 ( 2.1 ) |
| Eye allergy | 12 | 38.5 ( 21.82 - 67.92 ) | 38.46 ( 435.38 ) | 38.25 ( 21.68 ) | 5.26 ( 2.5 ) |
| Dyspraxia | 12 | 104.64 ( 59.15 - 185.11 ) | 104.53 ( 1211.88 ) | 102.96 ( 58.2 ) | 6.69 ( 2.73 ) |
| Foreign body in eye | 12 | 63.42 ( 35.91 - 111.99 ) | 63.35 ( 729.64 ) | 62.78 ( 35.55 ) | 5.97 ( 2.64 ) |
| Eyelid irritation | 12 | 25.75 ( 14.6 - 45.4 ) | 25.72 ( 284.06 ) | 25.63 ( 14.53 ) | 4.68 ( 2.34 ) |
| Eyelid disorder | 11 | 25.26 ( 13.97 - 45.68 ) | 25.24 ( 255.16 ) | 25.15 ( 13.91 ) | 4.65 ( 2.23 ) |
| Reaction to excipient | 11 | 11.2 ( 6.2 - 20.24 ) | 11.19 ( 101.92 ) | 11.17 ( 6.18 ) | 3.48 ( 1.76 ) |
| Symptom recurrence | 11 | 12.23 ( 6.76 - 22.09 ) | 12.21 ( 113.07 ) | 12.19 ( 6.75 ) | 3.61 ( 1.82 ) |
| Inability to afford medication | 11 | 6.23 ( 3.45 - 11.25 ) | 6.22 ( 48.16 ) | 6.22 ( 3.44 ) | 2.64 ( 1.28 ) |
| Ear discomfort | 10 | 4.94 ( 2.66 - 9.18 ) | 4.93 ( 31.36 ) | 4.93 ( 2.65 ) | 2.3 ( 0.99 ) |
| Initial insomnia | 10 | 5.42 ( 2.92 - 10.08 ) | 5.42 ( 36.01 ) | 5.42 ( 2.91 ) | 2.44 ( 1.08 ) |
| Therapy change | 10 | 5.03 ( 2.71 - 9.36 ) | 5.03 ( 32.27 ) | 5.03 ( 2.7 ) | 2.33 ( 1.01 ) |
| Claustrophobia | 10 | 47.99 ( 25.76 - 89.41 ) | 47.95 ( 456.51 ) | 47.62 ( 25.56 ) | 5.57 ( 2.31 ) |
| Deposit eye | 10 | 181.34 ( 96.75 - 339.9 ) | 181.19 ( 1745.43 ) | 176.51 ( 94.17 ) | 7.46 ( 2.5 ) |
| Vitreous floaters | 10 | 5.37 ( 2.89 - 9.99 ) | 5.37 ( 35.5 ) | 5.36 ( 2.88 ) | 2.42 ( 1.07 ) |
| Instillation site swelling | 10 | 268.79 ( 142.84 - 505.79 ) | 268.56 ( 2564.33 ) | 258.39 ( 137.32 ) | 8.01 ( 2.52 ) |
| Therapeutic product effect delayed | 10 | 5.13 ( 2.76 - 9.54 ) | 5.13 ( 33.2 ) | 5.12 ( 2.76 ) | 2.36 ( 1.03 ) |
| Eye infection | 9 | 4.13 ( 2.15 - 7.95 ) | 4.13 ( 21.34 ) | 4.13 ( 2.15 ) | 2.05 ( 0.74 ) |
| Blepharitis | 9 | 12.59 ( 6.54 - 24.21 ) | 12.58 ( 95.74 ) | 12.56 ( 6.53 ) | 3.65 ( 1.63 ) |
| Substance abuse | 9 | 5.31 ( 2.76 - 10.21 ) | 5.31 ( 31.45 ) | 5.3 ( 2.76 ) | 2.41 ( 0.98 ) |
| Product colour issue | 9 | 9.01 ( 4.68 - 17.32 ) | 9 ( 63.92 ) | 8.99 ( 4.67 ) | 3.17 ( 1.41 ) |
| Personality change | 9 | 5.1 ( 2.65 - 9.81 ) | 5.1 ( 29.65 ) | 5.1 ( 2.65 ) | 2.35 ( 0.94 ) |
| Corneal graft rejection | 9 | 261.53 ( 134.36 - 509.06 ) | 261.33 ( 2247.6 ) | 251.69 ( 129.31 ) | 7.98 ( 2.34 ) |
| Therapeutic product effect variable | 9 | 9.42 ( 4.9 - 18.13 ) | 9.42 ( 67.63 ) | 9.41 ( 4.89 ) | 3.23 ( 1.44 ) |
| Apnoea | 8 | 5.53 ( 2.76 - 11.06 ) | 5.52 ( 29.62 ) | 5.52 ( 2.76 ) | 2.46 ( 0.91 ) |
| Corneal abrasion | 8 | 30.1 ( 15.03 - 60.3 ) | 30.08 ( 223.93 ) | 29.95 ( 14.95 ) | 4.9 ( 1.86 ) |
| Logorrhoea | 8 | 11.68 ( 5.84 - 23.37 ) | 11.67 ( 77.93 ) | 11.65 ( 5.82 ) | 3.54 ( 1.45 ) |
| Drug tolerance | 8 | 8.13 ( 4.06 - 16.27 ) | 8.13 ( 49.94 ) | 8.12 ( 4.06 ) | 3.02 ( 1.22 ) |
| Eyelid pain | 8 | 23.48 ( 11.72 - 47.01 ) | 23.46 ( 171.44 ) | 23.38 ( 11.68 ) | 4.55 ( 1.78 ) |
| Sinus headache | 8 | 7.09 ( 3.54 - 14.19 ) | 7.09 ( 41.79 ) | 7.08 ( 3.54 ) | 2.82 ( 1.12 ) |
| Bradyphrenia | 8 | 5.93 ( 2.97 - 11.87 ) | 5.93 ( 32.76 ) | 5.93 ( 2.96 ) | 2.57 ( 0.97 ) |
| Product deposit | 8 | 27.14 ( 13.55 - 54.36 ) | 27.12 ( 200.5 ) | 27.02 ( 13.49 ) | 4.76 ( 1.83 ) |
| Dacryostenosis acquired | 8 | 34.49 ( 17.21 - 69.11 ) | 34.47 ( 258.68 ) | 34.3 ( 17.12 ) | 5.1 ( 1.9 ) |
| Cystoid macular oedema | 8 | 22.24 ( 11.11 - 44.53 ) | 22.22 ( 161.62 ) | 22.15 ( 11.06 ) | 4.47 ( 1.76 ) |
| Transcription medication error | 8 | 24.23 ( 12.1 - 48.52 ) | 24.21 ( 177.4 ) | 24.13 ( 12.05 ) | 4.59 ( 1.79 ) |
| Reaction to preservatives | 8 | 49.45 ( 24.66 - 99.15 ) | 49.42 ( 376.75 ) | 49.07 ( 24.47 ) | 5.62 ( 1.99 ) |
| Sensation of foreign body | 8 | 5.2 ( 2.6 - 10.41 ) | 5.2 ( 27.11 ) | 5.2 ( 2.6 ) | 2.38 ( 0.86 ) |
| Liquid product physical issue | 8 | 6.76 ( 3.38 - 13.52 ) | 6.75 ( 39.16 ) | 6.75 ( 3.37 ) | 2.75 ( 1.08 ) |
| Iritis | 7 | 12.16 ( 5.79 - 25.52 ) | 12.15 ( 71.49 ) | 12.13 ( 5.78 ) | 3.6 ( 1.32 ) |
| Tachyphrenia | 7 | 9.92 ( 4.72 - 20.82 ) | 9.91 ( 56.02 ) | 9.9 ( 4.72 ) | 3.31 ( 1.21 ) |
| Ear disorder | 7 | 7.68 ( 3.66 - 16.13 ) | 7.68 ( 40.63 ) | 7.67 ( 3.66 ) | 2.94 ( 1.04 ) |
| Dissociation | 7 | 5.76 ( 2.75 - 12.1 ) | 5.76 ( 27.52 ) | 5.76 ( 2.74 ) | 2.53 ( 0.83 ) |
| Catatonia | 7 | 7.16 ( 3.41 - 15.02 ) | 7.15 ( 37.02 ) | 7.15 ( 3.41 ) | 2.84 ( 0.99 ) |
| Ulcerative keratitis | 7 | 13.3 ( 6.33 - 27.93 ) | 13.29 ( 79.43 ) | 13.27 ( 6.32 ) | 3.73 ( 1.37 ) |
| Product formulation issue | 7 | 4.73 ( 2.25 - 9.93 ) | 4.73 ( 20.58 ) | 4.73 ( 2.25 ) | 2.24 ( 0.67 ) |
| Instillation site foreign body sensation | 7 | 220.33 ( 103.77 - 467.8 ) | 220.2 ( 1479.47 ) | 213.32 ( 100.47 ) | 7.74 ( 1.91 ) |
| Instillation site pruritus | 7 | 80.94 ( 38.41 - 170.56 ) | 80.89 ( 545.82 ) | 79.95 ( 37.94 ) | 6.32 ( 1.85 ) |
| Anal haemorrhage | 6 | 11.11 ( 4.99 - 24.76 ) | 11.11 ( 55.1 ) | 11.09 ( 4.98 ) | 3.47 ( 1.09 ) |
| Pulse abnormal | 6 | 12.7 ( 5.7 - 28.29 ) | 12.69 ( 64.51 ) | 12.67 ( 5.69 ) | 3.66 ( 1.16 ) |
| Skin tightness | 6 | 6.35 ( 2.85 - 14.15 ) | 6.35 ( 27.03 ) | 6.35 ( 2.85 ) | 2.67 ( 0.76 ) |
| Corneal scar | 6 | 66.98 ( 29.97 - 149.7 ) | 66.94 ( 385.95 ) | 66.3 ( 29.66 ) | 6.05 ( 1.59 ) |
| Eye operation | 6 | 7.38 ( 3.31 - 16.43 ) | 7.37 ( 33.02 ) | 7.37 ( 3.31 ) | 2.88 ( 0.86 ) |
| Ischaemia | 6 | 6.86 ( 3.08 - 15.28 ) | 6.86 ( 30 ) | 6.85 ( 3.08 ) | 2.78 ( 0.81 ) |
| Keratitis | 6 | 10.88 ( 4.88 - 24.24 ) | 10.87 ( 53.71 ) | 10.86 ( 4.87 ) | 3.44 ( 1.08 ) |
| Homicidal ideation | 6 | 8.41 ( 3.77 - 18.72 ) | 8.4 ( 39.08 ) | 8.39 ( 3.77 ) | 3.07 ( 0.94 ) |
| Respiration abnormal | 6 | 4.62 ( 2.08 - 10.3 ) | 4.62 ( 17.02 ) | 4.62 ( 2.07 ) | 2.21 ( 0.51 ) |
| Conjunctivitis allergic | 6 | 23.35 ( 10.47 - 52.05 ) | 23.34 ( 127.84 ) | 23.26 ( 10.43 ) | 4.54 ( 1.38 ) |
| Prostatic disorder | 6 | 5.5 ( 2.47 - 12.24 ) | 5.49 ( 22.03 ) | 5.49 ( 2.46 ) | 2.46 ( 0.65 ) |
| Drug screen negative | 6 | 15.6 ( 7 - 34.77 ) | 15.6 ( 81.78 ) | 15.56 ( 6.98 ) | 3.96 ( 1.24 ) |
| Eye colour change | 6 | 27.45 ( 12.31 - 61.21 ) | 27.43 ( 152.22 ) | 27.33 ( 12.26 ) | 4.77 ( 1.43 ) |
| Micturition disorder | 6 | 8.81 ( 3.95 - 19.61 ) | 8.8 ( 41.44 ) | 8.79 ( 3.95 ) | 3.14 ( 0.96 ) |
| Ocular hypertension | 6 | 17.05 ( 7.65 - 38 ) | 17.04 ( 90.39 ) | 17 ( 7.63 ) | 4.09 ( 1.28 ) |
| Gluten sensitivity | 6 | 19.02 ( 8.53 - 42.38 ) | 19.01 ( 102.07 ) | 18.96 ( 8.5 ) | 4.24 ( 1.32 ) |
| Therapeutic product ineffective for unapproved indication | 6 | 65.05 ( 29.11 - 145.39 ) | 65.02 ( 374.64 ) | 64.41 ( 28.82 ) | 6.01 ( 1.58 ) |
| Product contamination physical | 6 | 13.32 ( 5.98 - 29.67 ) | 13.31 ( 68.18 ) | 13.29 ( 5.96 ) | 3.73 ( 1.18 ) |
| Product solubility abnormal | 6 | 5.77 ( 2.59 - 12.85 ) | 5.77 ( 23.63 ) | 5.76 ( 2.59 ) | 2.53 ( 0.69 ) |
| Gastrointestinal motility disorder | 6 | 5.78 ( 2.59 - 12.86 ) | 5.77 ( 23.66 ) | 5.77 ( 2.59 ) | 2.53 ( 0.69 ) |
| Vascular injury | 5 | 18.2 ( 7.57 - 43.8 ) | 18.2 ( 81.04 ) | 18.15 ( 7.54 ) | 4.18 ( 1.05 ) |
| Acute psychosis | 5 | 10.54 ( 4.39 - 25.36 ) | 10.54 ( 43.11 ) | 10.53 ( 4.38 ) | 3.4 ( 0.84 ) |
| Morbid thoughts | 5 | 23.59 ( 9.8 - 56.76 ) | 23.58 ( 107.72 ) | 23.5 ( 9.76 ) | 4.55 ( 1.13 ) |
| Hypopnoea | 5 | 7.18 ( 2.99 - 17.26 ) | 7.18 ( 26.56 ) | 7.17 ( 2.98 ) | 2.84 ( 0.64 ) |
| Corneal disorder | 5 | 12.37 ( 5.14 - 29.75 ) | 12.37 ( 52.15 ) | 12.35 ( 5.13 ) | 3.63 ( 0.91 ) |
| Derealisation | 5 | 12.44 ( 5.17 - 29.91 ) | 12.43 ( 52.46 ) | 12.41 ( 5.16 ) | 3.63 ( 0.92 ) |
| Conjunctival haemorrhage | 5 | 6.64 ( 2.76 - 15.97 ) | 6.64 ( 23.93 ) | 6.63 ( 2.76 ) | 2.73 ( 0.6 ) |
| Cataract subcapsular | 5 | 44.78 ( 18.58 - 107.92 ) | 44.76 ( 212.52 ) | 44.47 ( 18.45 ) | 5.47 ( 1.25 ) |
| Intraocular pressure test abnormal | 5 | 37.97 ( 15.76 - 91.48 ) | 37.96 ( 178.93 ) | 37.75 ( 15.67 ) | 5.24 ( 1.22 ) |
| Instillation site dryness | 5 | 188.82 ( 77.63 - 459.25 ) | 188.74 ( 908.5 ) | 183.67 ( 75.51 ) | 7.52 ( 1.35 ) |
| Product closure removal difficult | 5 | 25.34 ( 10.53 - 61 ) | 25.33 ( 116.44 ) | 25.24 ( 10.49 ) | 4.66 ( 1.14 ) |
| Abnormal sleep-related event | 5 | 19.59 ( 8.14 - 47.13 ) | 19.58 ( 87.91 ) | 19.53 ( 8.12 ) | 4.29 ( 1.08 ) |
| Superficial injury of eye | 5 | 23.59 ( 9.8 - 56.76 ) | 23.58 ( 107.72 ) | 23.5 ( 9.76 ) | 4.55 ( 1.13 ) |
| Glare | 4 | 41.57 ( 15.55 - 111.11 ) | 41.56 ( 157.37 ) | 41.31 ( 15.46 ) | 5.37 ( 0.89 ) |
| Ear swelling | 4 | 9.24 ( 3.47 - 24.65 ) | 9.24 ( 29.36 ) | 9.23 ( 3.46 ) | 3.21 ( 0.51 ) |
| Biliary colic | 4 | 6.38 ( 2.39 - 17.01 ) | 6.38 ( 18.12 ) | 6.37 ( 2.39 ) | 2.67 ( 0.33 ) |
| Tooth hypoplasia | 4 | 125.87 ( 46.81 - 338.47 ) | 125.83 ( 486.34 ) | 123.56 ( 45.95 ) | 6.95 ( 0.97 ) |
| Echolalia | 4 | 70.07 ( 26.16 - 187.68 ) | 70.05 ( 269.47 ) | 69.34 ( 25.89 ) | 6.12 ( 0.94 ) |
| Breast abscess | 4 | 20.46 ( 7.67 - 54.6 ) | 20.45 ( 73.78 ) | 20.39 ( 7.64 ) | 4.35 ( 0.77 ) |
| Anxiety disorder | 4 | 6.32 ( 2.37 - 16.84 ) | 6.31 ( 17.87 ) | 6.31 ( 2.37 ) | 2.66 ( 0.32 ) |
| Reading disorder | 4 | 8.87 ( 3.33 - 23.65 ) | 8.86 ( 27.87 ) | 8.85 ( 3.32 ) | 3.15 ( 0.49 ) |
| Gun shot wound | 4 | 14.71 ( 5.51 - 39.25 ) | 14.71 ( 50.99 ) | 14.68 ( 5.5 ) | 3.88 ( 0.68 ) |
| Halo vision | 4 | 18.25 ( 6.84 - 48.69 ) | 18.24 ( 65.01 ) | 18.19 ( 6.82 ) | 4.19 ( 0.74 ) |
| Agoraphobia | 4 | 15.22 ( 5.71 - 40.61 ) | 15.22 ( 53.02 ) | 15.19 ( 5.69 ) | 3.92 ( 0.69 ) |
| Emotional poverty | 4 | 11.19 ( 4.2 - 29.85 ) | 11.19 ( 37.05 ) | 11.17 ( 4.19 ) | 3.48 ( 0.59 ) |
| Polymyalgia rheumatica | 4 | 7.31 ( 2.74 - 19.49 ) | 7.31 ( 21.76 ) | 7.3 ( 2.74 ) | 2.87 ( 0.4 ) |
| Visceral congestion | 4 | 42.75 ( 15.99 - 114.27 ) | 42.73 ( 162.01 ) | 42.47 ( 15.89 ) | 5.41 ( 0.9 ) |
| Peripheral nerve injury | 4 | 23.97 ( 8.98 - 64 ) | 23.97 ( 87.73 ) | 23.89 ( 8.95 ) | 4.58 ( 0.8 ) |
| Depression suicidal | 4 | 6.08 ( 2.28 - 16.2 ) | 6.08 ( 16.95 ) | 6.07 ( 2.28 ) | 2.6 ( 0.3 ) |
| Congestive hepatopathy | 4 | 17.13 ( 6.42 - 45.71 ) | 17.13 ( 60.59 ) | 17.09 ( 6.4 ) | 4.09 ( 0.73 ) |
| Terminal insomnia | 4 | 11.72 ( 4.4 - 31.27 ) | 11.72 ( 39.15 ) | 11.7 ( 4.39 ) | 3.55 ( 0.61 ) |
| Paradoxical drug reaction | 4 | 5.72 ( 2.14 - 15.24 ) | 5.71 ( 15.55 ) | 5.71 ( 2.14 ) | 2.51 ( 0.26 ) |
| Acquired immunodeficiency syndrome | 4 | 49.79 ( 18.62 - 133.17 ) | 49.78 ( 189.8 ) | 49.42 ( 18.48 ) | 5.63 ( 0.91 ) |
| Intraocular pressure test | 4 | 92.48 ( 34.47 - 248.08 ) | 92.44 ( 356.97 ) | 91.22 ( 34 ) | 6.51 ( 0.96 ) |
| Cyclitis | 3 | 453.09 ( 140.79 - 1458.19 ) | 452.98 ( 1268.38 ) | 424.73 ( 131.97 ) | 8.73 ( 0.49 ) |
| Corneal oedema | 3 | 6.92 ( 2.23 - 21.47 ) | 6.92 ( 15.18 ) | 6.91 ( 2.23 ) | 2.79 ( 0.04 ) |
| Eyelid rash | 3 | 17.67 ( 5.69 - 54.87 ) | 17.66 ( 47.04 ) | 17.62 ( 5.67 ) | 4.14 ( 0.33 ) |
| Sexual abuse | 3 | 38.25 ( 12.3 - 119 ) | 38.24 ( 108.2 ) | 38.04 ( 12.23 ) | 5.25 ( 0.44 ) |
| Legal problem | 3 | 25.3 ( 8.14 - 78.61 ) | 25.29 ( 69.73 ) | 25.2 ( 8.11 ) | 4.66 ( 0.39 ) |
| Bone graft | 3 | 17.92 ( 5.77 - 55.64 ) | 17.91 ( 47.78 ) | 17.87 ( 5.75 ) | 4.16 ( 0.33 ) |
| Sleep deficit | 3 | 8.94 ( 2.88 - 27.74 ) | 8.94 ( 21.12 ) | 8.93 ( 2.88 ) | 3.16 ( 0.14 ) |
| Hypervigilance | 3 | 16.13 ( 5.19 - 50.09 ) | 16.13 ( 42.47 ) | 16.09 ( 5.18 ) | 4.01 ( 0.31 ) |
| Imprisonment | 3 | 7.84 ( 2.53 - 24.33 ) | 7.84 ( 17.88 ) | 7.83 ( 2.52 ) | 2.97 ( 0.09 ) |
| Transient global amnesia | 3 | 20.59 ( 6.63 - 63.98 ) | 20.59 ( 55.75 ) | 20.53 ( 6.61 ) | 4.36 ( 0.36 ) |
| Dark circles under eyes | 3 | 10.02 ( 3.23 - 31.11 ) | 10.02 ( 24.33 ) | 10.01 ( 3.22 ) | 3.32 ( 0.18 ) |
| Hepatosplenomegaly | 3 | 6.6 ( 2.13 - 20.49 ) | 6.6 ( 14.24 ) | 6.6 ( 2.13 ) | 2.72 ( 0.02 ) |
| Hypoaesthesia eye | 3 | 25.97 ( 8.36 - 80.72 ) | 25.97 ( 71.74 ) | 25.87 ( 8.32 ) | 4.69 ( 0.39 ) |
| Salivary gland pain | 3 | 73.08 ( 23.42 - 228 ) | 73.06 ( 210.96 ) | 72.29 ( 23.17 ) | 6.18 ( 0.49 ) |
| Finger amputation | 3 | 21.19 ( 6.82 - 65.84 ) | 21.19 ( 57.53 ) | 21.13 ( 6.8 ) | 4.4 ( 0.36 ) |
| Contact lens intolerance | 3 | 112.03 ( 35.79 - 350.65 ) | 112 ( 324.68 ) | 110.2 ( 35.21 ) | 6.78 ( 0.5 ) |
| Hallucination, olfactory | 3 | 38.18 ( 12.27 - 118.78 ) | 38.17 ( 107.99 ) | 37.96 ( 12.2 ) | 5.25 ( 0.44 ) |
| Eyelid exfoliation | 3 | 20.27 ( 6.52 - 62.96 ) | 20.26 ( 54.77 ) | 20.21 ( 6.5 ) | 4.34 ( 0.35 ) |
| Adjustment disorder | 3 | 12.91 ( 4.16 - 40.09 ) | 12.91 ( 32.9 ) | 12.89 ( 4.15 ) | 3.69 ( 0.25 ) |
| Herpes ophthalmic | 3 | 11.26 ( 3.63 - 34.95 ) | 11.26 ( 27.99 ) | 11.24 ( 3.62 ) | 3.49 ( 0.21 ) |
| Instillation site haemorrhage | 3 | 351.54 ( 110.13 - 1122.1 ) | 351.45 ( 996.8 ) | 334.21 ( 104.7 ) | 8.38 ( 0.5 ) |
| Intraocular pressure decreased | 3 | 27.4 ( 8.82 - 85.18 ) | 27.4 ( 76 ) | 27.29 ( 8.78 ) | 4.77 ( 0.4 ) |
| Suspected product tampering | 3 | 31.61 ( 10.17 - 98.29 ) | 31.6 ( 88.49 ) | 31.46 ( 10.12 ) | 4.98 ( 0.42 ) |
| Anterior chamber inflammation | 3 | 27.74 ( 8.92 - 86.22 ) | 27.73 ( 76.99 ) | 27.62 ( 8.89 ) | 4.79 ( 0.4 ) |
| Antiphospholipid antibodies positive | 3 | 28.92 ( 9.3 - 89.9 ) | 28.91 ( 80.5 ) | 28.8 ( 9.26 ) | 4.85 ( 0.41 ) |
| Incorrect product dosage form administered | 3 | 15.09 ( 4.86 - 46.86 ) | 15.09 ( 39.38 ) | 15.06 ( 4.85 ) | 3.91 ( 0.29 ) |
| Depersonalisation/derealisation disorder | 3 | 6.57 ( 2.12 - 20.39 ) | 6.57 ( 14.15 ) | 6.56 ( 2.12 ) | 2.71 ( 0.01 ) |
| Congenital tracheomalacia | 3 | 194.18 ( 61.62 - 611.92 ) | 194.13 ( 560.41 ) | 188.77 ( 59.9 ) | 7.56 ( 0.51 ) |
| Jessner's lymphocytic infiltration | 3 | 755.15 ( 229.06 - 2489.59 ) | 754.96 ( 2033.01 ) | 679.57 ( 206.13 ) | 9.41 ( 0.46 ) |
| Suspected product quality issue | 3 | 10.36 ( 3.34 - 32.16 ) | 10.36 ( 25.32 ) | 10.34 ( 3.33 ) | 3.37 ( 0.19 ) |
| Patient dissatisfaction with treatment | 3 | 9.23 ( 2.97 - 28.64 ) | 9.23 ( 21.98 ) | 9.22 ( 2.97 ) | 3.2 ( 0.15 ) |

Abbreviation: ROR, reporting odds ratio; PRR, proportional reporting ratio; EBGM, empirical Bayesian geometric mean; EBGM05, the lower limit of the 95% CI of EBGM; IC, information component; IC025, the lower limit of the 95% CI of the IC; CI, confidence interval; PT,preferred term.

**Supplementary Table S4**. Top 50 most frequent adverse events for temazepam at the preferred term (PT) level in males from FAERS data.

| **PT** | **Case numbers** | **ROR(95%CI)** | **PRR(χ^2^)** | **EBGM(EBGM05)** | **IC(IC025)** |
| --- | --- | --- | --- | --- | --- |
| Toxicity to various agents | 96 | 13.36 ( 10.89 - 16.39 ) | 12.84 ( 1050.2 ) | 12.82 ( 10.45 ) | 3.68 ( 3.21 ) |
| Completed suicide | 53 | 14.84 ( 11.3 - 19.49 ) | 14.52 ( 666.77 ) | 14.49 ( 11.03 ) | 3.86 ( 3.14 ) |
| Insomnia | 43 | 4.61 ( 3.41 - 6.23 ) | 4.54 ( 119.12 ) | 4.54 ( 3.36 ) | 2.18 ( 1.63 ) |
| Drug abuse | 37 | 7.98 ( 5.76 - 11.04 ) | 7.87 ( 221.95 ) | 7.86 ( 5.68 ) | 2.97 ( 2.26 ) |
| Drug interaction | 35 | 4.83 ( 3.46 - 6.75 ) | 4.77 ( 104.65 ) | 4.77 ( 3.42 ) | 2.25 ( 1.63 ) |
| Vision blurred | 32 | 7.3 ( 5.15 - 10.35 ) | 7.21 ( 171.43 ) | 7.21 ( 5.08 ) | 2.85 ( 2.09 ) |
| Eye pain | 25 | 17.99 ( 12.12 - 26.69 ) | 17.8 ( 395.75 ) | 17.76 ( 11.97 ) | 4.15 ( 2.86 ) |
| Eye irritation | 23 | 17.04 ( 11.3 - 25.71 ) | 16.88 ( 343.12 ) | 16.85 ( 11.17 ) | 4.07 ( 2.75 ) |
| Aggression | 22 | 7.27 ( 4.78 - 11.07 ) | 7.21 ( 117.82 ) | 7.21 ( 4.74 ) | 2.85 ( 1.9 ) |
| Intraocular pressure increased | 20 | 43.3 ( 27.84 - 67.32 ) | 42.93 ( 814.52 ) | 42.69 ( 27.45 ) | 5.42 ( 3.2 ) |
| Product quality issue | 16 | 3.38 ( 2.07 - 5.53 ) | 3.37 ( 26.64 ) | 3.36 ( 2.06 ) | 1.75 ( 0.86 ) |
| Visual impairment | 16 | 3.85 ( 2.35 - 6.3 ) | 3.83 ( 33.49 ) | 3.83 ( 2.34 ) | 1.94 ( 1.01 ) |
| Abnormal behaviour | 16 | 7.29 ( 4.46 - 11.92 ) | 7.25 ( 86.14 ) | 7.24 ( 4.43 ) | 2.86 ( 1.7 ) |
| Amnesia | 15 | 6.04 ( 3.63 - 10.04 ) | 6.01 ( 62.62 ) | 6 ( 3.61 ) | 2.59 ( 1.47 ) |
| Dysarthria | 15 | 9.16 ( 5.51 - 15.22 ) | 9.11 ( 108.2 ) | 9.1 ( 5.47 ) | 3.19 ( 1.87 ) |
| Withdrawal syndrome | 15 | 9.2 ( 5.54 - 15.3 ) | 9.15 ( 108.84 ) | 9.14 ( 5.5 ) | 3.19 ( 1.87 ) |
| Disturbance in attention | 13 | 6 ( 3.48 - 10.34 ) | 5.97 ( 53.76 ) | 5.96 ( 3.46 ) | 2.58 ( 1.36 ) |
| Therapeutic product effect decreased | 13 | 3.72 ( 2.15 - 6.41 ) | 3.7 ( 25.65 ) | 3.7 ( 2.14 ) | 1.89 ( 0.86 ) |
| Depressed level of consciousness | 13 | 7.88 ( 4.57 - 13.6 ) | 7.84 ( 77.61 ) | 7.84 ( 4.54 ) | 2.97 ( 1.62 ) |
| Eye swelling | 11 | 11.27 ( 6.23 - 20.39 ) | 11.22 ( 102.29 ) | 11.2 ( 6.19 ) | 3.49 ( 1.76 ) |
| Nervousness | 11 | 7.52 ( 4.16 - 13.61 ) | 7.49 ( 61.86 ) | 7.49 ( 4.14 ) | 2.9 ( 1.44 ) |
| Restlessness | 11 | 6.65 ( 3.67 - 12.02 ) | 6.62 ( 52.47 ) | 6.61 ( 3.66 ) | 2.73 ( 1.34 ) |
| Lacrimation increased | 11 | 12.01 ( 6.64 - 21.73 ) | 11.96 ( 110.35 ) | 11.94 ( 6.6 ) | 3.58 ( 1.81 ) |
| Photophobia | 10 | 17.68 ( 9.5 - 32.94 ) | 17.61 ( 156.36 ) | 17.57 ( 9.44 ) | 4.14 ( 1.94 ) |
| Psychotic disorder | 10 | 6.83 ( 3.67 - 12.72 ) | 6.81 ( 49.51 ) | 6.8 ( 3.65 ) | 2.77 ( 1.28 ) |
| Panic attack | 9 | 8.21 ( 4.26 - 15.8 ) | 8.18 ( 56.66 ) | 8.17 ( 4.24 ) | 3.03 ( 1.33 ) |
| Ocular hyperaemia | 9 | 6.15 ( 3.19 - 11.84 ) | 6.13 ( 38.62 ) | 6.12 ( 3.18 ) | 2.61 ( 1.1 ) |
| Thinking abnormal | 9 | 12.77 ( 6.63 - 24.59 ) | 12.72 ( 97.09 ) | 12.7 ( 6.6 ) | 3.67 ( 1.63 ) |
| Product substitution issue | 9 | 4.68 ( 2.43 - 9.01 ) | 4.66 ( 25.92 ) | 4.66 ( 2.42 ) | 2.22 ( 0.86 ) |
| Foreign body sensation in eyes | 9 | 57.93 ( 30.03 - 111.75 ) | 57.7 ( 497.71 ) | 57.27 ( 29.69 ) | 5.84 ( 2.19 ) |
| Dry eye | 8 | 6.97 ( 3.48 - 13.97 ) | 6.95 ( 40.76 ) | 6.95 ( 3.47 ) | 2.8 ( 1.1 ) |
| Tinnitus | 8 | 4.87 ( 2.43 - 9.74 ) | 4.85 ( 24.47 ) | 4.85 ( 2.42 ) | 2.28 ( 0.8 ) |
| Delirium | 8 | 4.63 ( 2.31 - 9.28 ) | 4.62 ( 22.7 ) | 4.62 ( 2.31 ) | 2.21 ( 0.76 ) |
| Nightmare | 8 | 5.74 ( 2.87 - 11.49 ) | 5.72 ( 31.16 ) | 5.72 ( 2.86 ) | 2.52 ( 0.94 ) |
| Product physical issue | 8 | 12.55 ( 6.26 - 25.14 ) | 12.51 ( 84.59 ) | 12.49 ( 6.23 ) | 3.64 ( 1.49 ) |
| Product prescribing error | 8 | 4.89 ( 2.44 - 9.79 ) | 4.88 ( 24.64 ) | 4.87 ( 2.43 ) | 2.28 ( 0.8 ) |
| Glaucoma | 7 | 11.2 ( 5.33 - 23.53 ) | 11.17 ( 64.72 ) | 11.15 ( 5.31 ) | 3.48 ( 1.27 ) |
| Poisoning | 7 | 12.07 ( 5.75 - 25.37 ) | 12.04 ( 70.77 ) | 12.02 ( 5.72 ) | 3.59 ( 1.31 ) |
| Coordination abnormal | 7 | 13.07 ( 6.22 - 27.47 ) | 13.04 ( 77.67 ) | 13.01 ( 6.19 ) | 3.7 ( 1.36 ) |
| Sedation | 6 | 5.34 ( 2.4 - 11.91 ) | 5.33 ( 21.11 ) | 5.33 ( 2.39 ) | 2.41 ( 0.63 ) |
| Eye pruritus | 6 | 7 ( 3.14 - 15.6 ) | 6.98 ( 30.74 ) | 6.98 ( 3.13 ) | 2.8 ( 0.82 ) |
| Photosensitivity reaction | 6 | 9.51 ( 4.27 - 21.2 ) | 9.49 ( 45.5 ) | 9.48 ( 4.25 ) | 3.24 ( 1.01 ) |
| Paranoia | 5 | 5.89 ( 2.45 - 14.17 ) | 5.88 ( 20.23 ) | 5.87 ( 2.44 ) | 2.55 ( 0.52 ) |
| Miosis | 5 | 11.73 ( 4.87 - 28.22 ) | 11.7 ( 48.87 ) | 11.69 ( 4.86 ) | 3.55 ( 0.89 ) |
| Claustrophobia | 5 | 119.36 ( 49.29 - 289.03 ) | 119.1 ( 576.44 ) | 117.26 ( 48.42 ) | 6.87 ( 1.33 ) |
| Bradykinesia | 5 | 18.36 ( 7.63 - 44.2 ) | 18.32 ( 81.7 ) | 18.28 ( 7.59 ) | 4.19 ( 1.05 ) |
| Bradyphrenia | 5 | 18.97 ( 7.88 - 45.67 ) | 18.93 ( 84.72 ) | 18.89 ( 7.84 ) | 4.24 ( 1.06 ) |
| Middle insomnia | 5 | 7.37 ( 3.06 - 17.74 ) | 7.36 ( 27.46 ) | 7.35 ( 3.06 ) | 2.88 ( 0.66 ) |
| Eye discharge | 5 | 17.28 ( 7.18 - 41.59 ) | 17.24 ( 76.33 ) | 17.2 ( 7.15 ) | 4.1 ( 1.04 ) |
| Logorrhoea | 5 | 28.77 ( 11.94 - 69.3 ) | 28.71 ( 133.22 ) | 28.6 ( 11.87 ) | 4.84 ( 1.17 ) |

Abbreviation: ROR, reporting odds ratio; PRR, proportional reporting ratio; EBGM, empirical Bayesian geometric mean; EBGM05, the lower limit of the 95% CI of EBGM; IC, information component; IC025, the lower limit of the 95% CI of the IC; CI, confidence interval; PT,preferred term.

**Supplementary Table S5**. Top 50 most frequent adverse events for temazepam at the preferred term (PT) level in females from FAERS data.

| **PT** | **Case numbers** | **ROR(95%CI)** | **PRR(χ^2^)** | **EBGM(EBGM05)** | **IC(IC025)** |
| --- | --- | --- | --- | --- | --- |
| Drug ineffective | 222 | 2.62 ( 2.29 - 2.99 ) | 2.53 ( 210.05 ) | 2.53 ( 2.21 ) | 1.34 ( 1.13 ) |
| Off label use | 132 | 2.55 ( 2.14 - 3.03 ) | 2.5 ( 119.97 ) | 2.5 ( 2.1 ) | 1.32 ( 1.05 ) |
| Eye irritation | 119 | 28.89 ( 24.07 - 34.67 ) | 28.11 ( 3101.74 ) | 28 ( 23.33 ) | 4.81 ( 4.25 ) |
| Vision blurred | 96 | 9.4 ( 7.68 - 11.51 ) | 9.21 ( 703.31 ) | 9.2 ( 7.51 ) | 3.2 ( 2.79 ) |
| Eye pain | 88 | 21.24 ( 17.19 - 26.24 ) | 20.82 ( 1657.13 ) | 20.76 ( 16.8 ) | 4.38 ( 3.78 ) |
| Product use in unapproved indication | 78 | 5.87 ( 4.69 - 7.34 ) | 5.78 ( 309.1 ) | 5.78 ( 4.62 ) | 2.53 ( 2.12 ) |
| Ocular hyperaemia | 75 | 21.95 ( 17.46 - 27.59 ) | 21.58 ( 1468.58 ) | 21.52 ( 17.12 ) | 4.43 ( 3.75 ) |
| Insomnia | 65 | 3.32 ( 2.6 - 4.25 ) | 3.29 ( 103.92 ) | 3.29 ( 2.57 ) | 1.72 ( 1.31 ) |
| Completed suicide | 65 | 13.32 ( 10.42 - 17.02 ) | 13.13 ( 727.89 ) | 13.11 ( 10.26 ) | 3.71 ( 3.11 ) |
| Product quality issue | 47 | 4.68 ( 3.51 - 6.24 ) | 4.64 ( 134.28 ) | 4.63 ( 3.48 ) | 2.21 ( 1.69 ) |
| Eye swelling | 42 | 13.66 ( 10.08 - 18.52 ) | 13.54 ( 487.14 ) | 13.51 ( 9.97 ) | 3.76 ( 2.94 ) |
| Toxicity to various agents | 40 | 4.12 ( 3.02 - 5.62 ) | 4.09 ( 93.46 ) | 4.09 ( 2.99 ) | 2.03 ( 1.47 ) |
| Foreign body sensation in eyes | 39 | 78.19 ( 56.94 - 107.36 ) | 77.48 ( 2911.2 ) | 76.61 ( 55.8 ) | 6.26 ( 4.27 ) |
| Eye pruritus | 38 | 14.76 ( 10.72 - 20.32 ) | 14.64 ( 482.02 ) | 14.61 ( 10.61 ) | 3.87 ( 2.97 ) |
| Lacrimation increased | 37 | 16.91 ( 12.23 - 23.38 ) | 16.77 ( 547.62 ) | 16.73 ( 12.1 ) | 4.06 ( 3.09 ) |
| Visual impairment | 32 | 3.25 ( 2.3 - 4.6 ) | 3.23 ( 49.47 ) | 3.23 ( 2.28 ) | 1.69 ( 1.09 ) |
| Dry eye | 29 | 7.48 ( 5.19 - 10.77 ) | 7.43 ( 161.4 ) | 7.42 ( 5.15 ) | 2.89 ( 2.08 ) |
| Eye discharge | 28 | 35.28 ( 24.31 - 51.21 ) | 35.06 ( 921.8 ) | 34.88 ( 24.03 ) | 5.12 ( 3.47 ) |
| Product physical consistency issue | 26 | 96.85 ( 65.68 - 142.79 ) | 96.27 ( 2416.75 ) | 94.92 ( 64.38 ) | 6.57 ( 3.84 ) |
| Product prescribing error | 26 | 9.89 ( 6.72 - 14.55 ) | 9.84 ( 206.2 ) | 9.82 ( 6.68 ) | 3.3 ( 2.33 ) |
| Intraocular pressure increased | 24 | 29.5 ( 19.73 - 44.1 ) | 29.34 ( 654.26 ) | 29.22 ( 19.54 ) | 4.87 ( 3.2 ) |
| Drug ineffective for unapproved indication | 24 | 6.83 ( 4.57 - 10.2 ) | 6.79 ( 118.55 ) | 6.79 ( 4.54 ) | 2.76 ( 1.88 ) |
| Therapeutic product effect decreased | 22 | 3.22 ( 2.12 - 4.89 ) | 3.21 ( 33.45 ) | 3.21 ( 2.11 ) | 1.68 ( 0.94 ) |
| Burning sensation | 21 | 3.63 ( 2.37 - 5.58 ) | 3.62 ( 39.85 ) | 3.62 ( 2.36 ) | 1.86 ( 1.08 ) |
| Eye disorder | 19 | 7.47 ( 4.76 - 11.73 ) | 7.44 ( 105.93 ) | 7.44 ( 4.74 ) | 2.89 ( 1.84 ) |
| Ocular discomfort | 19 | 24.86 ( 15.83 - 39.04 ) | 24.75 ( 431.54 ) | 24.66 ( 15.7 ) | 4.62 ( 2.85 ) |
| Intentional overdose | 19 | 4.17 ( 2.66 - 6.55 ) | 4.16 ( 45.56 ) | 4.15 ( 2.65 ) | 2.05 ( 1.2 ) |
| Product substitution issue | 19 | 4.78 ( 3.05 - 7.5 ) | 4.76 ( 56.51 ) | 4.76 ( 3.03 ) | 2.25 ( 1.36 ) |
| Eyelid oedema | 18 | 18.32 ( 11.53 - 29.13 ) | 18.25 ( 292.76 ) | 18.2 ( 11.45 ) | 4.19 ( 2.59 ) |
| Eye inflammation | 16 | 25.25 ( 15.44 - 41.29 ) | 25.16 ( 369.83 ) | 25.07 ( 15.33 ) | 4.65 ( 2.67 ) |
| Withdrawal syndrome | 16 | 5.83 ( 3.57 - 9.52 ) | 5.81 ( 63.67 ) | 5.8 ( 3.55 ) | 2.54 ( 1.48 ) |
| Instillation site pain | 15 | 69.83 ( 41.95 - 116.23 ) | 69.58 ( 1003.66 ) | 68.88 ( 41.38 ) | 6.11 ( 2.99 ) |
| Product packaging quantity issue | 15 | 25.76 ( 15.5 - 42.81 ) | 25.67 ( 354.36 ) | 25.58 ( 15.39 ) | 4.68 ( 2.61 ) |
| Product residue present | 14 | 16.14 ( 9.55 - 27.3 ) | 16.09 ( 197.73 ) | 16.06 ( 9.5 ) | 4.01 ( 2.25 ) |
| Photophobia | 13 | 9.53 ( 5.53 - 16.43 ) | 9.5 ( 98.79 ) | 9.49 ( 5.5 ) | 3.25 ( 1.79 ) |
| Product physical issue | 13 | 9.11 ( 5.29 - 15.72 ) | 9.09 ( 93.5 ) | 9.08 ( 5.27 ) | 3.18 ( 1.75 ) |
| Instillation site erythema | 13 | 311.24 ( 178.35 - 543.14 ) | 310.3 ( 3831.37 ) | 296.67 ( 170 ) | 8.21 ( 2.95 ) |
| Instillation site irritation | 13 | 177.67 ( 102.35 - 308.42 ) | 177.14 ( 2218.49 ) | 172.62 ( 99.44 ) | 7.43 ( 2.92 ) |
| Product delivery mechanism issue | 13 | 41.2 ( 23.87 - 71.14 ) | 41.08 ( 505.34 ) | 40.84 ( 23.65 ) | 5.35 ( 2.63 ) |
| Somnambulism | 12 | 23.3 ( 13.21 - 41.1 ) | 23.24 ( 254.52 ) | 23.16 ( 13.13 ) | 4.53 ( 2.29 ) |
| Nightmare | 12 | 5.02 ( 2.85 - 8.85 ) | 5.01 ( 38.52 ) | 5.01 ( 2.84 ) | 2.32 ( 1.13 ) |
| Disease recurrence | 12 | 4.11 ( 2.33 - 7.24 ) | 4.1 ( 28.09 ) | 4.09 ( 2.32 ) | 2.03 ( 0.92 ) |
| Eyelids pruritus | 11 | 37.62 ( 20.78 - 68.1 ) | 37.53 ( 388.96 ) | 37.33 ( 20.62 ) | 5.22 ( 2.38 ) |
| Extra dose administered | 11 | 4.37 ( 2.42 - 7.9 ) | 4.36 ( 28.5 ) | 4.36 ( 2.41 ) | 2.12 ( 0.93 ) |
| Abnormal sensation in eye | 11 | 31.29 ( 17.29 - 56.63 ) | 31.22 ( 320.26 ) | 31.08 ( 17.17 ) | 4.96 ( 2.31 ) |
| Abnormal behaviour | 10 | 4.82 ( 2.59 - 8.97 ) | 4.81 ( 30.19 ) | 4.81 ( 2.59 ) | 2.27 ( 0.97 ) |
| Product prescribing issue | 10 | 9.7 ( 5.21 - 18.05 ) | 9.68 ( 77.76 ) | 9.67 ( 5.2 ) | 3.27 ( 1.56 ) |
| Conjunctivitis | 9 | 7.36 ( 3.82 - 14.15 ) | 7.34 ( 49.28 ) | 7.34 ( 3.81 ) | 2.88 ( 1.25 ) |
| Abnormal dreams | 9 | 4.72 ( 2.45 - 9.07 ) | 4.71 ( 26.28 ) | 4.71 ( 2.45 ) | 2.23 ( 0.87 ) |
| Product container issue | 9 | 17.43 ( 9.05 - 33.55 ) | 17.39 ( 138.71 ) | 17.35 ( 9.01 ) | 4.12 ( 1.8 ) |

Abbreviation: ROR, reporting odds ratio; PRR, proportional reporting ratio; EBGM, empirical Bayesian geometric mean; EBGM05, the lower limit of the 95% CI of EBGM; IC, information component; IC025, the lower limit of the 95% CI of the IC; CI, confidence interval; PT,preferred term.

**Supplementary Table S6**. Adverse events at the PT level for temazepam in patients aged under 18 from FAERS data.

| **PT** | **Case numbers** | **ROR(95%CI)** | **PRR(χ^2^)** | **EBGM(EBGM05)** | **IC(IC025)** |
| --- | --- | --- | --- | --- | --- |
| Seizure | 2 | 10.72 ( 2.53 - 45.49 ) | 9.95 ( 16.22 ) | 9.94 ( 2.34 ) | 3.31 ( -0.45 ) |
| Hypertensive crisis | 2 | 1085.79 ( 253.75 - 4646.06 ) | 999.01 ( 1968.11 ) | 985.96 ( 230.42 ) | 9.95 ( -0.19 ) |
| Toxicity to various agents | 2 | 14.98 ( 3.53 - 63.54 ) | 13.86 ( 24 ) | 13.86 ( 3.27 ) | 3.79 ( -0.38 ) |
| Glaucoma | 1 | 358.72 ( 48.31 - 2663.32 ) | 344.41 ( 340.89 ) | 342.85 ( 46.18 ) | 8.42 ( -1.13 ) |
| Overdose | 1 | 5.69 ( 0.77 - 42.1 ) | 5.51 ( 3.72 ) | 5.51 ( 0.74 ) | 2.46 ( -1.37 ) |
| Diplopia | 1 | 135.42 ( 18.29 - 1002.7 ) | 130.04 ( 127.87 ) | 129.82 ( 17.53 ) | 7.02 ( -1.13 ) |
| Fall | 1 | 24.2 ( 3.27 - 178.94 ) | 23.27 ( 21.35 ) | 23.27 ( 3.15 ) | 4.54 ( -1.18 ) |
| Facial paralysis | 1 | 149.9 ( 20.24 - 1110.09 ) | 143.94 ( 141.72 ) | 143.67 ( 19.4 ) | 7.17 ( -1.13 ) |
| Drug abuse | 1 | 21.85 ( 2.96 - 161.56 ) | 21.02 ( 19.09 ) | 21.01 ( 2.84 ) | 4.39 ( -1.19 ) |
| Off label use | 1 | 1.58 ( 0.21 - 11.66 ) | 1.55 ( 0.2 ) | 1.55 ( 0.21 ) | 0.64 ( -1.86 ) |
| Rebound effect | 1 | 170.02 ( 22.95 - 1259.42 ) | 163.26 ( 160.96 ) | 162.91 ( 21.99 ) | 7.35 ( -1.13 ) |
| Spinal disorder | 1 | 946.56 ( 126.58 - 7078.28 ) | 908.74 ( 895.98 ) | 897.93 ( 120.08 ) | 9.81 ( -1.14 ) |
| Poisoning | 1 | 117.75 ( 15.91 - 871.69 ) | 113.08 ( 110.96 ) | 112.91 ( 15.25 ) | 6.82 ( -1.13 ) |
| Depression | 1 | 11.86 ( 1.6 - 87.72 ) | 11.43 ( 9.55 ) | 11.43 ( 1.55 ) | 3.51 ( -1.24 ) |
| Panic attack | 1 | 72.98 ( 9.86 - 539.94 ) | 70.1 ( 68.09 ) | 70.03 ( 9.47 ) | 6.13 ( -1.14 ) |
| Drug ineffective | 1 | 2.04 ( 0.28 - 15.05 ) | 2 ( 0.51 ) | 2 ( 0.27 ) | 1 ( -1.72 ) |
| Open angle glaucoma | 1 | 6043.64 ( 760.37 - 48036.94 ) | 5801.94 ( 5385.7 ) | 5387.59 ( 677.83 ) | 12.4 ( -1.24 ) |
| Visual acuity reduced | 1 | 158.68 ( 21.42 - 1175.27 ) | 152.37 ( 150.12 ) | 152.07 ( 20.53 ) | 7.25 ( -1.13 ) |
| Ocular hypertension | 1 | 545.57 ( 73.31 - 4059.86 ) | 523.79 ( 518.23 ) | 520.18 ( 69.9 ) | 9.02 ( -1.13 ) |
| Product use in unapproved indication | 1 | 5.6 ( 0.76 - 41.38 ) | 5.41 ( 3.62 ) | 5.41 ( 0.73 ) | 2.44 ( -1.37 ) |
| Psychomotor hyperactivity | 1 | 30.61 ( 4.14 - 226.38 ) | 29.43 ( 27.49 ) | 29.42 ( 3.98 ) | 4.88 ( -1.17 ) |
| Intentional product misuse | 1 | 25.04 ( 3.39 - 185.13 ) | 24.07 ( 22.15 ) | 24.07 ( 3.25 ) | 4.59 ( -1.18 ) |

Abbreviation: ROR, reporting odds ratio; PRR, proportional reporting ratio; EBGM, empirical Bayesian geometric mean; EBGM05, the lower limit of the 95% CI of EBGM; IC, information component; IC025, the lower limit of the 95% CI of the IC; CI, confidence interval; PT,preferred term.

**Supplementary Table S7**. Adverse events at the PT level for temazepam in patients aged 18 to 65 from FAERS data.

| **PT** | **Case numbers** | **ROR(95%CI)** | **PRR(χ^2^)** | **EBGM(EBGM05)** | **IC(IC025)** |
| --- | --- | --- | --- | --- | --- |
| Death | 172 | 8.88 ( 7.61 - 10.36 ) | 8.41 ( 1129.01 ) | 8.4 ( 7.2 ) | 3.07 ( 2.78 ) |
| Toxicity to various agents | 122 | 11.7 ( 9.76 - 14.03 ) | 11.24 ( 1140.78 ) | 11.22 ( 9.36 ) | 3.49 ( 3.11 ) |
| Completed suicide | 76 | 11.82 ( 9.41 - 14.84 ) | 11.53 ( 731.3 ) | 11.51 ( 9.16 ) | 3.53 ( 3.01 ) |
| Drug abuse | 45 | 7.05 ( 5.25 - 9.46 ) | 6.95 ( 229.53 ) | 6.94 ( 5.17 ) | 2.8 ( 2.19 ) |
| Drug interaction | 44 | 5.88 ( 4.36 - 7.92 ) | 5.8 ( 175.28 ) | 5.8 ( 4.31 ) | 2.54 ( 1.96 ) |
| Eye irritation | 44 | 27.14 ( 20.14 - 36.58 ) | 26.74 ( 1086.76 ) | 26.64 ( 19.77 ) | 4.74 ( 3.65 ) |
| Vision blurred | 33 | 4.98 ( 3.53 - 7.01 ) | 4.93 ( 103.55 ) | 4.93 ( 3.5 ) | 2.3 ( 1.64 ) |
| Eye pain | 28 | 13.29 ( 9.16 - 19.29 ) | 13.17 ( 314.59 ) | 13.15 ( 9.06 ) | 3.72 ( 2.67 ) |
| Ocular hyperaemia | 27 | 14.43 ( 9.88 - 21.09 ) | 14.3 ( 333.66 ) | 14.28 ( 9.77 ) | 3.84 ( 2.73 ) |
| Overdose | 25 | 3.2 ( 2.16 - 4.75 ) | 3.18 ( 37.51 ) | 3.18 ( 2.15 ) | 1.67 ( 0.98 ) |
| Intentional overdose | 20 | 4.58 ( 2.95 - 7.12 ) | 4.56 ( 55.62 ) | 4.56 ( 2.93 ) | 2.19 ( 1.33 ) |
| Amnesia | 19 | 5.8 ( 3.69 - 9.1 ) | 5.77 ( 74.86 ) | 5.76 ( 3.67 ) | 2.53 ( 1.57 ) |
| Eye swelling | 18 | 9.93 ( 6.24 - 15.79 ) | 9.87 ( 143.42 ) | 9.86 ( 6.2 ) | 3.3 ( 2.08 ) |
| Intraocular pressure increased | 18 | 48.98 ( 30.76 - 77.97 ) | 48.67 ( 834.9 ) | 48.35 ( 30.37 ) | 5.6 ( 3.12 ) |
| Dysarthria | 17 | 8.21 ( 5.1 - 13.23 ) | 8.17 ( 106.92 ) | 8.16 ( 5.07 ) | 3.03 ( 1.86 ) |
| Aggression | 17 | 7.3 ( 4.53 - 11.76 ) | 7.26 ( 91.74 ) | 7.25 ( 4.5 ) | 2.86 ( 1.75 ) |
| Lacrimation increased | 17 | 14.21 ( 8.82 - 22.9 ) | 14.13 ( 207.12 ) | 14.11 ( 8.75 ) | 3.82 ( 2.35 ) |
| Cardio-respiratory arrest | 17 | 6.59 ( 4.09 - 10.62 ) | 6.56 ( 80.06 ) | 6.55 ( 4.07 ) | 2.71 ( 1.64 ) |
| Eye pruritus | 16 | 11.19 ( 6.84 - 18.3 ) | 11.13 ( 147.41 ) | 11.12 ( 6.8 ) | 3.47 ( 2.1 ) |
| Depressed level of consciousness | 16 | 8.12 ( 4.97 - 13.27 ) | 8.08 ( 99.2 ) | 8.07 ( 4.94 ) | 3.01 ( 1.81 ) |
| Poisoning | 15 | 15.61 ( 9.39 - 25.94 ) | 15.53 ( 203.59 ) | 15.5 ( 9.33 ) | 3.95 ( 2.3 ) |
| Foreign body sensation in eyes | 15 | 91.32 ( 54.81 - 152.18 ) | 90.85 ( 1316.19 ) | 89.72 ( 53.84 ) | 6.49 ( 3.05 ) |
| Suicide attempt | 14 | 3.54 ( 2.09 - 5.99 ) | 3.53 ( 25.38 ) | 3.53 ( 2.09 ) | 1.82 ( 0.85 ) |
| Abnormal behaviour | 14 | 8.94 ( 5.29 - 15.12 ) | 8.9 ( 98.12 ) | 8.89 ( 5.26 ) | 3.15 ( 1.79 ) |
| Withdrawal syndrome | 14 | 6.68 ( 3.95 - 11.3 ) | 6.66 ( 67.28 ) | 6.65 ( 3.93 ) | 2.73 ( 1.52 ) |
| Restlessness | 13 | 6.66 ( 3.86 - 11.49 ) | 6.63 ( 62.18 ) | 6.63 ( 3.84 ) | 2.73 ( 1.47 ) |
| Dry eye | 12 | 5.64 ( 3.2 - 9.95 ) | 5.62 ( 45.6 ) | 5.62 ( 3.19 ) | 2.49 ( 1.25 ) |
| Product substitution issue | 12 | 4.22 ( 2.4 - 7.45 ) | 4.21 ( 29.38 ) | 4.21 ( 2.39 ) | 2.07 ( 0.95 ) |
| Eye discharge | 11 | 28.26 ( 15.61 - 51.14 ) | 28.15 ( 286.97 ) | 28.05 ( 15.5 ) | 4.81 ( 2.27 ) |
| Photophobia | 10 | 9.71 ( 5.22 - 18.08 ) | 9.68 ( 77.76 ) | 9.67 ( 5.19 ) | 3.27 ( 1.56 ) |
| Product physical consistency issue | 10 | 83.89 ( 44.92 - 156.65 ) | 83.6 ( 806.67 ) | 82.64 ( 44.26 ) | 6.37 ( 2.42 ) |
| Somnambulism | 9 | 24.48 ( 12.71 - 47.14 ) | 24.4 ( 201.32 ) | 24.32 ( 12.63 ) | 4.6 ( 1.95 ) |
| Psychotic disorder | 9 | 5.2 ( 2.7 - 10 ) | 5.18 ( 30.39 ) | 5.18 ( 2.69 ) | 2.37 ( 0.95 ) |
| Sedation | 8 | 5.69 ( 2.84 - 11.4 ) | 5.68 ( 30.84 ) | 5.68 ( 2.84 ) | 2.51 ( 0.94 ) |
| Cataract | 8 | 5 ( 2.5 - 10 ) | 4.98 ( 25.48 ) | 4.98 ( 2.49 ) | 2.32 ( 0.82 ) |
| Glaucoma | 7 | 12.53 ( 5.97 - 26.33 ) | 12.51 ( 73.98 ) | 12.48 ( 5.94 ) | 3.64 ( 1.33 ) |
| Eyelid oedema | 7 | 10.3 ( 4.91 - 21.65 ) | 10.28 ( 58.59 ) | 10.27 ( 4.89 ) | 3.36 ( 1.23 ) |
| Bradyphrenia | 7 | 17.3 ( 8.23 - 36.35 ) | 17.26 ( 106.97 ) | 17.22 ( 8.19 ) | 4.11 ( 1.48 ) |
| Substance abuse | 7 | 12.53 ( 5.97 - 26.33 ) | 12.51 ( 73.98 ) | 12.48 ( 5.94 ) | 3.64 ( 1.33 ) |
| Accidental overdose | 7 | 5.03 ( 2.39 - 10.55 ) | 5.02 ( 22.5 ) | 5.01 ( 2.39 ) | 2.33 ( 0.72 ) |
| Hangover | 6 | 30.7 ( 13.76 - 68.5 ) | 30.63 ( 171.28 ) | 30.51 ( 13.67 ) | 4.93 ( 1.45 ) |
| Eye inflammation | 6 | 18.43 ( 8.26 - 41.09 ) | 18.39 ( 98.42 ) | 18.34 ( 8.23 ) | 4.2 ( 1.31 ) |
| Eye disorder | 6 | 5.16 ( 2.32 - 11.5 ) | 5.15 ( 20.06 ) | 5.15 ( 2.31 ) | 2.36 ( 0.6 ) |
| Personality change | 6 | 12.33 ( 5.53 - 27.49 ) | 12.31 ( 62.24 ) | 12.29 ( 5.51 ) | 3.62 ( 1.14 ) |
| Pulmonary congestion | 6 | 8.99 ( 4.03 - 20.04 ) | 8.97 ( 42.46 ) | 8.96 ( 4.02 ) | 3.16 ( 0.97 ) |
| Thinking abnormal | 6 | 5.82 ( 2.61 - 12.97 ) | 5.81 ( 23.87 ) | 5.8 ( 2.6 ) | 2.54 ( 0.69 ) |
| Mydriasis | 5 | 6.61 ( 2.75 - 15.91 ) | 6.6 ( 23.77 ) | 6.6 ( 2.74 ) | 2.72 ( 0.59 ) |
| Logorrhoea | 5 | 25.55 ( 10.61 - 61.54 ) | 25.51 ( 117.34 ) | 25.42 ( 10.56 ) | 4.67 ( 1.14 ) |
| Bradykinesia | 5 | 21.91 ( 9.1 - 52.76 ) | 21.88 ( 99.31 ) | 21.81 ( 9.06 ) | 4.45 ( 1.11 ) |
| Ocular discomfort | 5 | 12.38 ( 5.14 - 29.78 ) | 12.36 ( 52.11 ) | 12.34 ( 5.13 ) | 3.62 ( 0.91 ) |

Abbreviation: ROR, reporting odds ratio; PRR, proportional reporting ratio; EBGM, empirical Bayesian geometric mean; EBGM05, the lower limit of the 95% CI of EBGM; IC, information component; IC025, the lower limit of the 95% CI of the IC; CI, confidence interval; PT,preferred term.

**Supplementary Table S8**. Adverse events at the PT level for temazepam in patients aged over 65 from FAERS data.

| **PT** | **Case numbers** | **ROR(95%CI)** | **PRR(χ^2^)** | **EBGM(EBGM05)** | **IC(IC025)** |
| --- | --- | --- | --- | --- | --- |
| Drug ineffective | 90 | 2.85 ( 2.31 - 3.51 ) | 2.78 ( 103.63 ) | 2.77 ( 2.25 ) | 1.47 ( 1.14 ) |
| Vision blurred | 69 | 14.49 ( 11.4 - 18.41 ) | 14.09 ( 838.32 ) | 14.05 ( 11.05 ) | 3.81 ( 3.21 ) |
| Eye pain | 67 | 34.45 ( 27 - 43.96 ) | 33.5 ( 2099.5 ) | 33.27 ( 26.07 ) | 5.06 ( 4.14 ) |
| Eye irritation | 67 | 37.27 ( 29.21 - 47.57 ) | 36.24 ( 2280.44 ) | 35.97 ( 28.19 ) | 5.17 ( 4.21 ) |
| Product use in unapproved indication | 46 | 6.7 ( 5.01 - 8.98 ) | 6.59 ( 218.52 ) | 6.58 ( 4.92 ) | 2.72 ( 2.13 ) |
| Completed suicide | 43 | 21.04 ( 15.55 - 28.47 ) | 20.67 ( 802.26 ) | 20.59 ( 15.22 ) | 4.36 ( 3.39 ) |
| Insomnia | 40 | 4.69 ( 3.43 - 6.41 ) | 4.62 ( 113.86 ) | 4.62 ( 3.38 ) | 2.21 ( 1.63 ) |
| Ocular hyperaemia | 40 | 25.96 ( 18.97 - 35.51 ) | 25.53 ( 938.52 ) | 25.4 ( 18.57 ) | 4.67 ( 3.54 ) |
| Visual impairment | 27 | 5.44 ( 3.72 - 7.95 ) | 5.38 ( 96.5 ) | 5.38 ( 3.68 ) | 2.43 ( 1.67 ) |
| Eye swelling | 26 | 22.39 ( 15.2 - 32.98 ) | 22.15 ( 522.92 ) | 22.05 ( 14.97 ) | 4.46 ( 3.07 ) |
| Product quality issue | 21 | 5.12 ( 3.33 - 7.86 ) | 5.08 ( 68.84 ) | 5.07 ( 3.3 ) | 2.34 ( 1.48 ) |
| Foreign body sensation in eyes | 20 | 70.17 ( 45.04 - 109.3 ) | 69.57 ( 1332.75 ) | 68.6 ( 44.04 ) | 6.1 ( 3.39 ) |
| Eye pruritus | 19 | 17.8 ( 11.32 - 27.98 ) | 17.67 ( 297.77 ) | 17.6 ( 11.2 ) | 4.14 ( 2.62 ) |
| Product prescribing error | 18 | 9.72 ( 6.11 - 15.47 ) | 9.66 ( 139.53 ) | 9.64 ( 6.06 ) | 3.27 ( 2.06 ) |
| Dry eye | 17 | 11.57 ( 7.18 - 18.66 ) | 11.5 ( 162.64 ) | 11.47 ( 7.12 ) | 3.52 ( 2.17 ) |
| Eye discharge | 16 | 40.71 ( 24.84 - 66.69 ) | 40.43 ( 610.35 ) | 40.11 ( 24.48 ) | 5.33 ( 2.9 ) |
| Lacrimation increased | 16 | 13.19 ( 8.06 - 21.59 ) | 13.11 ( 178.59 ) | 13.08 ( 7.99 ) | 3.71 ( 2.23 ) |
| Therapeutic product effect decreased | 16 | 7.37 ( 4.51 - 12.06 ) | 7.33 ( 87.41 ) | 7.32 ( 4.48 ) | 2.87 ( 1.71 ) |
| Intraocular pressure increased | 16 | 26.68 ( 16.29 - 43.68 ) | 26.5 ( 390.62 ) | 26.36 ( 16.1 ) | 4.72 ( 2.7 ) |
| Ocular discomfort | 12 | 34.38 ( 19.46 - 60.75 ) | 34.21 ( 384.19 ) | 33.97 ( 19.23 ) | 5.09 ( 2.46 ) |
| Tinnitus | 11 | 8.16 ( 4.51 - 14.75 ) | 8.12 ( 68.62 ) | 8.11 ( 4.48 ) | 3.02 ( 1.51 ) |
| Eye disorder | 11 | 8.04 ( 4.44 - 14.55 ) | 8.01 ( 67.39 ) | 8 ( 4.42 ) | 3 ( 1.5 ) |
| Disturbance in attention | 11 | 8.94 ( 4.94 - 16.18 ) | 8.91 ( 77.1 ) | 8.89 ( 4.91 ) | 3.15 ( 1.59 ) |
| Product physical consistency issue | 11 | 55.68 ( 30.69 - 101.02 ) | 55.42 ( 581.24 ) | 54.81 ( 30.21 ) | 5.78 ( 2.48 ) |
| Drug ineffective for unapproved indication | 11 | 6.07 ( 3.36 - 10.98 ) | 6.05 ( 46.33 ) | 6.04 ( 3.34 ) | 2.59 ( 1.25 ) |
| Nervousness | 10 | 4.87 ( 2.62 - 9.07 ) | 4.86 ( 30.62 ) | 4.85 ( 2.61 ) | 2.28 ( 0.97 ) |
| Withdrawal syndrome | 10 | 15.24 ( 8.18 - 28.39 ) | 15.18 ( 132.09 ) | 15.14 ( 8.13 ) | 3.92 ( 1.85 ) |
| Agitation | 9 | 3.96 ( 2.06 - 7.63 ) | 3.95 ( 19.85 ) | 3.95 ( 2.05 ) | 1.98 ( 0.69 ) |
| Photophobia | 9 | 19.39 ( 10.06 - 37.36 ) | 19.32 ( 155.75 ) | 19.25 ( 9.99 ) | 4.27 ( 1.85 ) |
| Nightmare | 9 | 8.88 ( 4.61 - 17.09 ) | 8.85 ( 62.56 ) | 8.83 ( 4.59 ) | 3.14 ( 1.39 ) |
| Burning sensation | 9 | 3.9 ( 2.03 - 7.51 ) | 3.89 ( 19.31 ) | 3.89 ( 2.02 ) | 1.96 ( 0.68 ) |
| Aggression | 8 | 7.32 ( 3.65 - 14.66 ) | 7.3 ( 43.42 ) | 7.29 ( 3.64 ) | 2.87 ( 1.14 ) |
| Eyelid oedema | 8 | 19.09 ( 9.52 - 38.28 ) | 19.03 ( 136.17 ) | 18.96 ( 9.46 ) | 4.25 ( 1.7 ) |
| Instillation site pain | 8 | 62.26 ( 30.96 - 125.19 ) | 62.05 ( 474.44 ) | 61.27 ( 30.47 ) | 5.94 ( 2.02 ) |
| Product packaging quantity issue | 8 | 21.9 ( 10.92 - 43.91 ) | 21.83 ( 158.29 ) | 21.73 ( 10.84 ) | 4.44 ( 1.75 ) |
| Instillation site irritation | 8 | 186.48 ( 91.93 - 378.28 ) | 185.84 ( 1416.34 ) | 179 ( 88.24 ) | 7.48 ( 2.12 ) |
| Product delivery mechanism issue | 8 | 63.58 ( 31.62 - 127.87 ) | 63.37 ( 484.75 ) | 62.56 ( 31.11 ) | 5.97 ( 2.03 ) |
| Irritability | 7 | 7.12 ( 3.39 - 14.96 ) | 7.1 ( 36.66 ) | 7.09 ( 3.38 ) | 2.83 ( 0.99 ) |
| Panic attack | 7 | 12.11 ( 5.76 - 25.46 ) | 12.08 ( 70.98 ) | 12.05 ( 5.73 ) | 3.59 ( 1.32 ) |
| Treatment noncompliance | 7 | 4.77 ( 2.27 - 10.02 ) | 4.76 ( 20.77 ) | 4.75 ( 2.26 ) | 2.25 ( 0.67 ) |
| Product use complaint | 7 | 10.82 ( 5.15 - 22.74 ) | 10.79 ( 62.05 ) | 10.77 ( 5.12 ) | 3.43 ( 1.25 ) |
| Instillation site erythema | 7 | 311.23 ( 144.78 - 669.05 ) | 310.31 ( 2027.96 ) | 291.64 ( 135.67 ) | 8.19 ( 1.91 ) |
| Abnormal sensation in eye | 7 | 47.05 ( 22.32 - 99.16 ) | 46.91 ( 311.53 ) | 46.47 ( 22.05 ) | 5.54 ( 1.77 ) |
| Product prescribing issue | 7 | 9.38 ( 4.46 - 19.71 ) | 9.36 ( 52.16 ) | 9.34 ( 4.44 ) | 3.22 ( 1.17 ) |
| Middle insomnia | 6 | 8.35 ( 3.75 - 18.62 ) | 8.33 ( 38.67 ) | 8.32 ( 3.73 ) | 3.06 ( 0.93 ) |
| Abnormal behaviour | 6 | 6.25 ( 2.8 - 13.93 ) | 6.23 ( 26.34 ) | 6.23 ( 2.79 ) | 2.64 ( 0.74 ) |
| Product residue present | 6 | 15.3 ( 6.86 - 34.14 ) | 15.27 ( 79.75 ) | 15.22 ( 6.82 ) | 3.93 ( 1.23 ) |
| Product container issue | 6 | 27.19 ( 12.17 - 60.71 ) | 27.12 ( 150.11 ) | 26.97 ( 12.08 ) | 4.75 ( 1.42 ) |
| Drug effective for unapproved indication | 6 | 10.58 ( 4.74 - 23.59 ) | 10.55 ( 51.79 ) | 10.53 ( 4.72 ) | 3.4 ( 1.06 ) |
| Claustrophobia | 5 | 147.63 ( 60.58 - 359.75 ) | 147.31 ( 705.12 ) | 142.99 ( 58.68 ) | 7.16 ( 1.34 ) |

Abbreviation: ROR, reporting odds ratio; PRR, proportional reporting ratio; EBGM, empirical Bayesian geometric mean; EBGM05, the lower limit of the 95% CI of EBGM; IC, information component; IC025, the lower limit of the 95% CI of the IC; CI, confidence interval; PT,preferred term.
